# Biodegradable Zinc‐Based Alloys for Guided Bone Regeneration Membranes: Feasibility, Current Status, and Future Prospects

**DOI:** 10.1002/advs.202506513

**Published:** 2025-09-17

**Authors:** Kai Chen, Ping Li, Qiang Guan, Xuenan Gu, Li Zhao, Linjun Huang, Chenyang Huang, Yu Qin, Chunhao Yu, Ting Zhang, Hejia Li, Yongcan Huang, Yufeng Zheng

**Affiliations:** ^1^ School of Materials Science and Engineering Peking University Beijing 100871 China; ^2^ Key Laboratory of Biomechanics and Mechanobiology (Beihang University) Ministry of Education Beijing Advanced Innovation Center for Biomedical Engineering School of Biological Science and Medical Engineering Beihang University Beijing 100083 China; ^3^ Shenzhen Engineering Laboratory of Orthopaedic Regenerative Technologies Department of Spine Surgery Peking University Shenzhen Hospital Shenzhen Peking University‐The Hong Kong University of Science and Technology Medical Center Shenzhen Guangdong Province 518036 China; ^4^ Department of Third Dental Center Peking University School and Hospital of Stomatology Beijing 100191 China; ^5^ Department of Prosthodontics School and Hospital of Stomatology Guangzhou Medical University Guangzhou Guangdong 510182 China; ^6^ Institute for Advanced Materials and Technology University of Science and Technology Beijing Beijing 100083 China; ^7^ School of Life, Beijing Institute of Technology No. 5, Zhongguancun South Street, Haidian District Beijing 100081 China; ^8^ Faculty of Advanced Science and Technology Kumamoto University 2‐39‐1 Kurokami Chuo‐Ku Kumamoto 860‐8555 Japan

**Keywords:** biodegradable zinc, current status, feasibility, future prospects, guided bone regeneration (GBR) membrane

## Abstract

The guided bone regeneration (GBR) technique is an effective method for treating inadequate alveolar ridge bone mass. The choice of barrier membrane materials plays a crucial role in the success of this technique. Recently, biodegradable zinc (Zn)‐based metallic barrier membranes have been extensively investigated as a novel option for alveolar bone defect repair. Although in vitro and animal studies using Zn‐based GBR membranes have shown some promising results, it remains uncertain whether these successes can be replicated in humans. In this review article, the clinical requirements for GBR membranes are discussed and the feasibility of Zn‐based alloys as a potential new option is assessed. Current advancements in the development of Zn‐based GBR membranes through alloying, surface modification, composite methods, and additive manufacturing techniques are also summarized. Importantly, several challenges persist, including stress corrosion, creep, and the need to balance osteogenesis with antimicrobial efficacy, which must be addressed in future studies. Overall, Zn‐based barrier membranes represent a biodegradable and multifunctional solution for enhancing bone regeneration in dental applications.

## Graphical abstract

Recent research has shown that biodegradable metals based on zinc (Zn) may be used as guided bone regeneration (GBR) membrane materials. This study examines their viability from the standpoints of material science and biology, reports on the state of research on Zn‐based GBR membranes, and suggests specific development plans based on the potential and challenges that now exist.

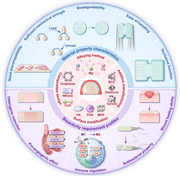

## 1 Introduction

**Figure 1:**
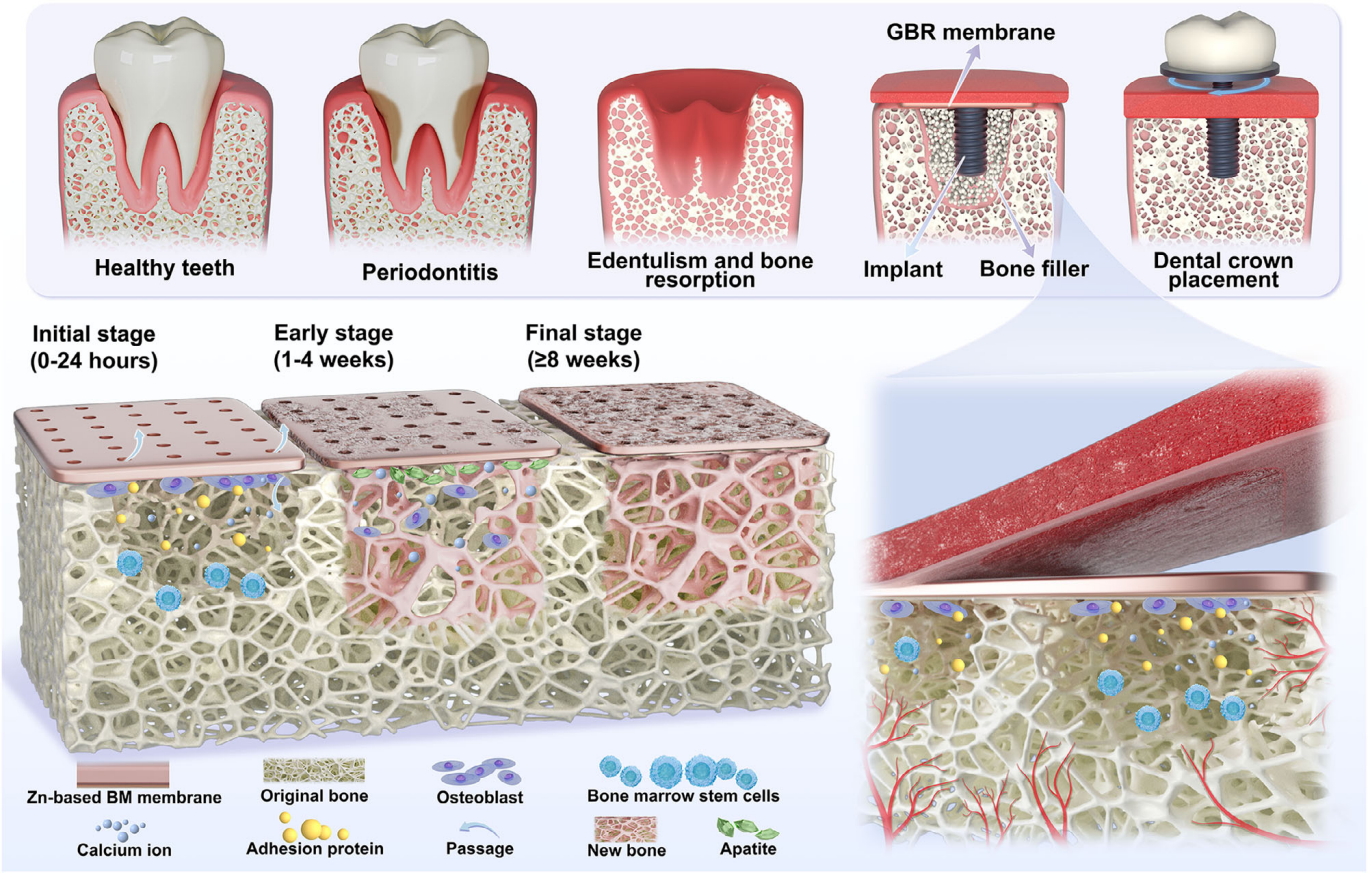
Schematic illustration demonstrating GBR implementation using bone graft material with biodegradable Zn‐based BM barrier membrane, alongside the proposed tissue regeneration mechanism mediated by the membranes that synergistically act as structural barriers and bioactive interfaces. Initial stage (0–24 h post‐trauma):‌ The membrane facilitates calcium ion absorption, osteoblast recruitment, and bone matrix deposition, initiating early osteogenesis through cellular activation. Early stage (1–4 weeks):‌ Accelerated mineralization drives apatite formation, with nascent bone emerging simultaneously at peripheral and central defect regions. Final stage (≥ 8 weeks):‌ Complete defect regeneration occurs through development of organized lamellar bone architecture, demonstrating structural and functional restoration.

The most prevalent oral illness in clinical practice is periodontitis, which is caused by inflammation and infection.^[^ ^1^ ^]^ Recently, the Centers for Disease Control and Prevention estimated that the prevalence of periodontal disease is 70.1% among individuals of 65 and older, while 47.2% of adults aged 30 and older have the condition.^[^ ^2^ ^]^ As periodontitis advances, the teeth may become looser and eventually fall out due to soft tissue retraction, alveolar bone resorption, and loss of supporting tissues. The most widely used method in clinical setting for tackling alveolar ridge bone mass is guided bone regeneration (GBR).^[^ ^3^ ^]^ GBR typically involves the use of grafting materials and a barrier membrane. The barrier helps create a gap between the soft tissue and the bone defect area, preventing the rapid growth of fibroblasts and epithelial cells while allowing osteoblasts with osteogenic potential to enter the area. This approach aims to facilitate effective bone regeneration (as shown in **Figure** **1**).^[^ ^4^ ^]^ The overall properties of the barrier membrane materials are likely to significantly influence the success of the GBR operation.

Currently, there are two primary categories of barrier membranes utilized in therapeutic settings: nonresorbable and resorbable membranes, as illustrated in **Table** **1**. Nonresorbable membranes, such as polytetrafluoroethylene (PTFE) and titanium membranes, offer the advantages of adequate mechanical support and spatial stability.^[^ ^5^ ^]^ However, their use is limited by the challenges, such as the need for secondary surgery for removal, high stiffness, significant morbidity, and the increased risk of membrane exposure. On the other hand, resorbable membranes are primarily consist of natural materials (such as collagen, extracellular matrices, chitosan, and alginate) and synthetic polymers (including polylactic acid (PLA), polyglycolic acid (PGA), and Poly‐DL‐lactide (PDLLA)).^[^ ^6^ ^]^ Nevertheless, resorbable membranes are also constrained by weak mechanical properties and variability in resorption rates. Additionally, the degradation products of synthetic polymer membranes pose risks of infection and transmission.^[^ ^7^ ^]^ As a result, there is an urgent need for new materials that are strong, biocompatible, and sufficiently malleable to be used as GBR membranes.

**Table 1:** Featured characteristics of the nonresorbable and resorbable GBR membranes.

Degradability	Technics	Materials	Types	Commercial name	Degradation period	Advantages	Disadvantages	Refs.
Nonresorbable	Synthetic	Polytetrafluoroethylene (PTFE)	Expanded PTFE Dense PTFE Titanium‐reinforced PTFE	Gore‐Tex Cytoplast TXT‐200 NeoGen Ti‐reinforced Gore‐Tex‐Ti Cytoplast Ti‐250		Steady mechanical support, strong barrier function, stable space‐making capability, assistance in stabilizing blood clots, and reasonable cost.	Second surgery removal, high risk of membrane exposure, high stiffness, and higher morbidity.	[5]
	Metallic	Titanium	Titanium mesh	FriosBoneShields Ridege‐Form Mesh				[12]
Resorbable	Natural	Noncross‐linked Collagen	Type I collagen Type I and III collagen Type I, III, IV, and other proteins	CollaTape; Tutodent BioGide; botiss Jason DynaMatrix	≈8 weeks ≈24 weeks 12–24 weeks	Better patient connection, enhanced biocompatibility, and no need for a second surgery.	Inadequate mechanical support, an unpredictable pace of decay, and the potential to collapse.	[13]
			Collagen with intermingled elastin	Creos xenoprotect	24–32 weeks			
		Cross‐linked Collagen	Cross‐linked type I collagen	BioMend^®^; OSSIX^®^ PLUS; OsseoGuard^®^	8–32 weeks			[14]
			Cross‐linked type I and type III	OsseoGuard Flex; EZ Cure	24–32 weeks			
	Synthetic	Aliphatic polyesters	Poly‐DL‐lactide (PDLLA)	Atrisorb	36–48 weeks	Better soft tissue regeneration	Restricted uses, the potential for infection by products, and the risk of disease transmission.	[15]
			Polylactic acid (PLA)	Epi‐Guide	24–48 weeks			[16]
			polyglycolic acid (PGA)	Biofix	24–48 weeks			
			Poly‐L‐lactide‐co glycolide (PLGA)	Resolut adapt	≈8 weeks			[5a]
	Metallic	Mg and its alloys	Pure Mg	NOVAMag	8–16 weeks	Good biocompatibility, sound osteoinductivity, low density and elastic modulus (close to bone), and MRI compatibility.	Rapid degradation rate, hydrogen evolution effect, high SCC susceptibility, and insufficient mechanical integrity.	[17]
		Zn and its alloys	Pure Zn		12–24 weeks (depending its thickness)	Good biocompatibility, suitable degradation rate, good malleability, intrinsic bacteriostatic behavior, osteogenic activity, steady mechanical support, strong barrier function, and MRI compatibility.	Age hardening, insufficient bacteriostatic activity against complex oral environment, inadequate osteogenic activity.	[18]

Recently, biodegradable metals (BMs) have emerged as promising options for barrier membranes in dental implantology, particularly those based on magnesium (Mg) and zinc (Zn). The term “BMs” refers to metals expected to corrode gradually in vivo, eliciting a suitable host response through the release of corrosion products that can be metabolized or assimilated by cells and/or tissue before completely dissolving. This occurs once their role in promoting tissue healing is fulfilled, without leaving behind implant residues.^[^ ^8^ ^]^ As shown in Table 1, resorbable metallic barrier membranes can provide adequate mechanical support and spatial stability after fulfilling their bone regeneration task, combining the merits of both resorbable and nonresorbable membranes. Initially, Mg and its alloys were considered for the fabrication of GBR membranes. However, their application has been limited by several issues, including rapid degradation rates, hydrogen gas generation during degradation, and high susceptibility to stress corrosion cracking (SCC).^[^ ^9^ ^]^ Additionally, Fe‐based BMs are deemed unsuitable for barrier membrane materials due to their slow degradation rate and the prolonged presence of degradation products in the body, which can lead to suboptimal biocompatibility with bone tissue, particularly given that iron primarily exists in the bloodstream.^[^ ^10^ ^]^ As a result, researchers have recently shifted their focus to biodegradable Zn‐based metals. Zn and its alloys, which exhibit moderate degradation rate (14–30 µm year^−1^), align well with the service life of barrier membranes (16–24 weeks) and do not produce gas during degradation, making them a promising alternative.^[^ ^3^, ^11^ ^]^ To date, there has been insufficient systematic analysis specially targeting Zn‐based biodegradable barrier membranes. Moreover, comprehensive assessments of their feasibility in physiological conditions and future development trajectories are still lacking.

This study first defines the essential performance criteria for ideal GBR membranes, considering both clinical requirements and practical implications. Our review then provides a focused examination of the material characteristics, corrosion behavior, and biological compatibility of Zn‐based membranes, while also analyzing their interactions with the alveolar ridge regeneration microenvironment. Then, this work employed a literature search strategy targeting the core concepts of “biodegradable Zn” and “guided bone regeneration membrane,” with systematic screening executed via the MEDLINE database. Through a critical synthesis of a decade's worth of research on Zn‐based membranes, we identify current challenges, highlight advantages, and outline strategic directions for future membrane design. The ultimate objective of this work is to integrate existing research findings and propose promising pathways for advancing Zn‐based barrier membrane technologies.

## 2 Clinical Design Requirements of GBR Membrane

The clinical applications of GBR technology in dental implant restoration primarily focus on three key areas: augmenting preimplant bone volume in the surgical site, repairing bone defects arising from immediate implant placement, and addressing pathological bone resorption caused by peri‐implantitis following implant surgery.^[^ ^19^ ^]^ To ensure predictable clinical efficacy in GBR, Wang et al. first propose the “PASS” principle‐an acronym encapsulating four foundational tenets: ‌P‌rimary wound closure (achieving a hermetic seal of the surgical site to facilitate undisturbed healing); ‌A‌ngiogenesis (stimulating neo‐vascularization to support metabolic demands of regenerating bone); ‌S‌pace creation/maintenance (ensuring volumetric sufficiency and structural integrity for osteogenesis); and ‌S‌tability (preserving the immobility of both the nascent blood clot and implant fixture to avert micromotion‐induced failure).^[^ ^20^ ^]^ This principle establishes a biomechanical and biological framework critical for successful GBR outcomes.

**Figure 2:**
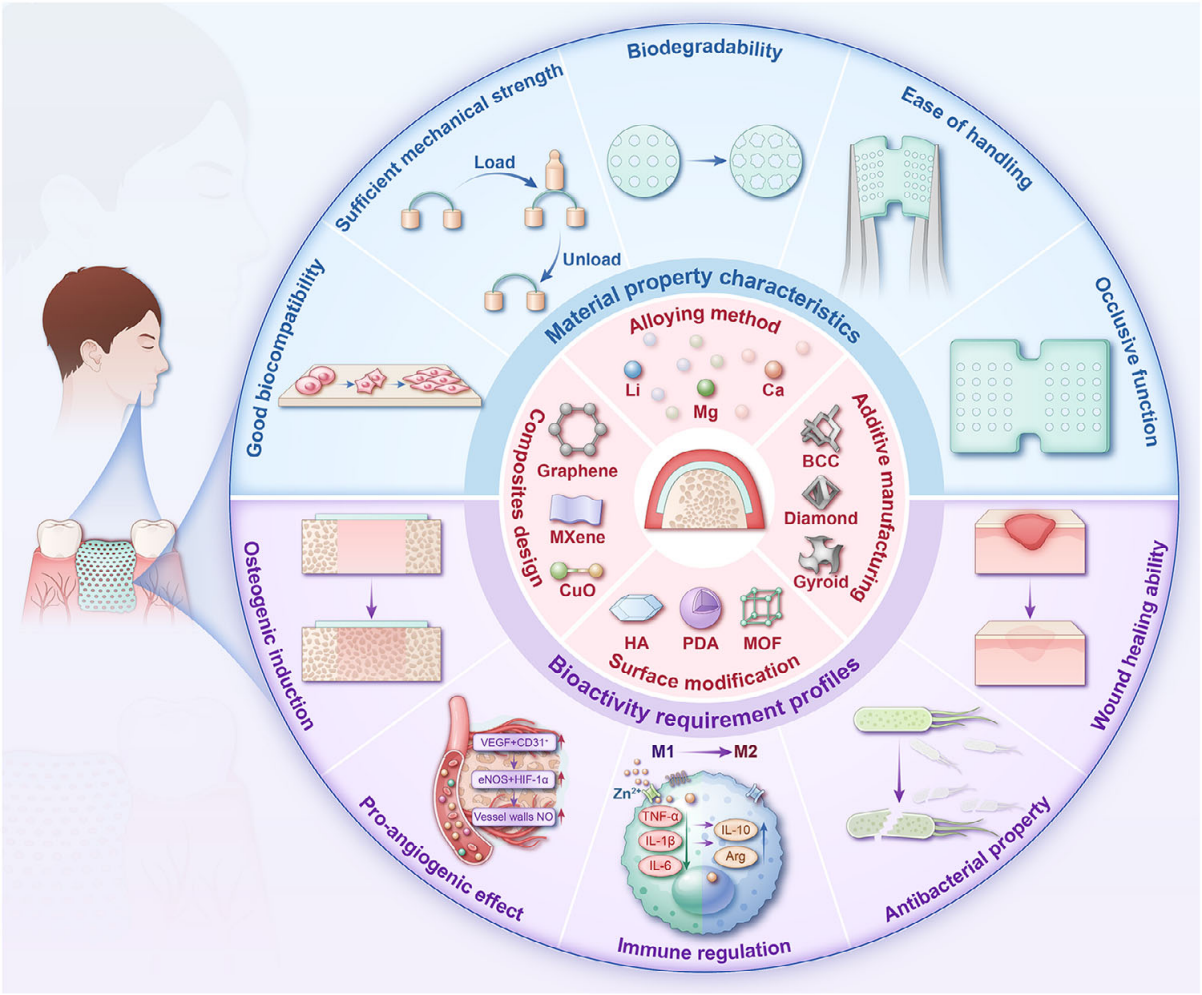
The materials property characteristics and bioactivity requirement profiles of an ideal GBR membrane.

Based on the biological principles outlined above, an ideal barrier membrane material should exhibit the following characteristics, as illustrated in **Figure** **2**: 1) ‌Good biocompatibility‌: The material should elicit appropriate host responses after implantation and contribute to tissue repair. It must not disrupt the surrounding tissues, impede osteoblast growth, or interfere with the healing process. 2) ‌Moderate mechanical strength‌: The membrane should possess adequate mechanical strength to maintain osteogenic space with spatial and temporal stability, providing mechanical integrity for at least 16–24 weeks,^[^ ^3b^ ^]^ which aligns with the alveolar bone regeneration cycle. 3) ‌Biodegradability‌: The material should degrade gradually and be safely absorbed after tissue reconstruction, avoiding the need for secondary surgical removal.^[^ ^8a^ ^]^ 4) ‌Ease of intraoperative handling‌: The membrane should be easily maneuverable during surgical procedures to accommodate various implantation sites and pathological conditions. Additionally, it should not be excessively rigid, as too much rigidity can compromise tissue integration or cause soft tissue dehiscence.^[^ ^21^ ^]^ 5) ‌Occlusive function‌: The membrane should prevent cellular invasion from the mucosa into the defect area while allowing for oxygen and nutrient exchange. Optimal fluid transport is essential for promoting metabolic activity and tissue regeneration, thereby enhancing osteogenic efficiency.^[^ ^22^ ^]^ 6) ‌Enhanced bioactivity‌: The material should support tissue healing through mechanisms, such as osteogenic induction, proangiogenic effects, immune regulation, and antibacterial properties.^[^ ^4a^ ^]^ Particularly, the capability to maintain spatial stability in the bone defect area and the barrier function are the most critical properties for a GBR membrane.

## 3 Feasibility of Zn‐Based Alloys as GBR Membranes

In recent years, biodegradable Zn‐based metallic barrier membranes have emerged as a promising solution for alveolar bone defect repair. Their superior mechanical properties and controlled degradation rates enable gradual dissolution following bone regeneration, addressing the limitations of current commercial barrier membranes. Known as a “revolutionary metallic biomaterial,” medical Zn alloys have the potential to replace existing commercial membranes in dental applications. However, the development of Zn‐based barrier membranes remains in its early stages. This analysis evaluates their feasibility as GBR membranes through several critical aspects.

### 3.1 Favorable Cytocompatibility and Hemocompatibility to Surrounding Tissues

**Figure 3:**
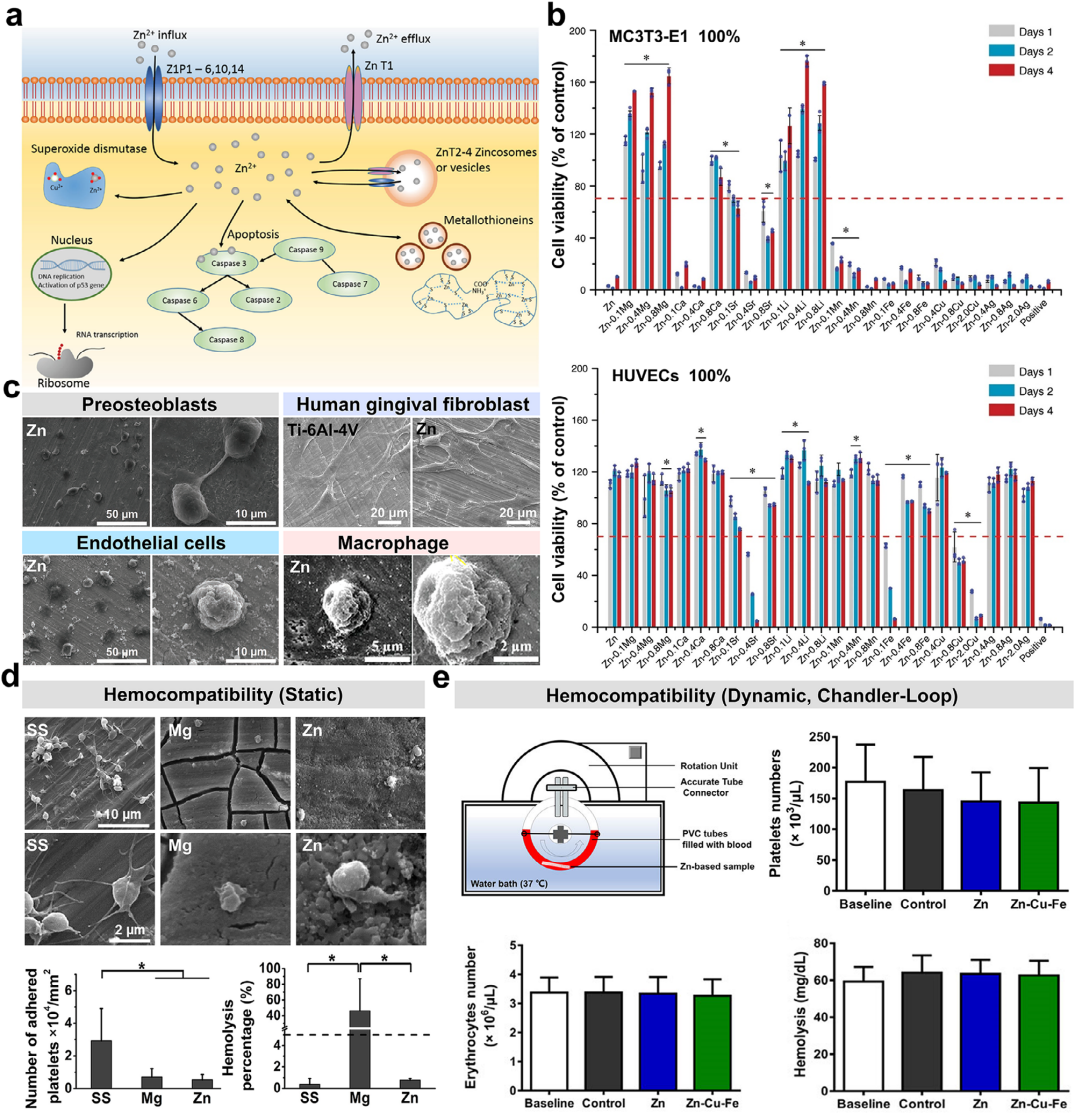
The cytocompatibility and hemocompatibility of Zn as GBR membrane. a) The biological functions of Zn in cells. Reproduced with permission.^[^ ^36^ ^]^ Copyright 2016, Wiley‐VCH. b) The cytocompatibility of Zn and its alloys towards MC3T3‐E1 cells and HUVECs, as evaluated through culture in 100% material extracts, was meticulously assessed. Reproduced with permission.^[^ ^11^ ^]^ Copyright 2020, Springer Nature. c) The adhesion patterns of four representative cell types (preosteoblasts (MC3T3‐E1), endothelial cells (HUVECs), human gingival fibroblasts (HGF), and macrophages (RAW264.7)) associated with osteogenic processes in GBR were characterized on pure Zn surfaces. Reproduced with permission.^[^ ^29^ ^]^ Copyright 2019, Wiley‐VCH. Reproduced with permission. Copyright 2024, Wiley‐VCH. Reproduced with permission. Copyright 2021, The Royal Society of Chemistry. d) The hemocompatibility of pure Zn under static immersion tests was evaluated, including surface platelet adhesion morphology, density, and hemolysis rate, in comparison to stainless steel (SS) and WE43 alloy (Mg). Reproduced with permission.^[^ ^34^ ^]^ Copyright 2019, Elsevier. e) The hemocompatibility of Zn and Zn‐Cu‐Fe alloys under dynamic testing conditions was evaluated via the Chandler‐Loop in vitro dynamic blood circulation model, with quantitative analysis of platelets numbers, erythrocytes count, and hemolysis extent. Reproduced with permission.^[^ ^35a^ ^]^ Copyright 2022, Frontiers.

From a physiological perspective, Zn is the second most abundant trace element in living organisms and plays a crucial role in cell biology, human anatomy, and physiology.^[^ ^23^ ^]^ Literature reports indicate that adults should consume 8–11 mg of Zn daily, with its serum concentration maintained at ≈12.4–17.4 µm.^[^ ^24^ ^]^ As illustrated in **Figure** **3**a, Zn is tightly regulated through channels in cell membranes, and once Zn^2+^ enters the cytoplasm, it performs several important functions, including regulating DNA replication, coordinating apoptosis, and activating metalloenzymes.^[^ ^3b^ ^]^ As a result, Zn plays a pivotal role in preventing and treating various pathophysiological conditions, such as cardiovascular, skeletal, and neurological disorders, as well as cancers.^[^ ^25^ ^]^ A deficiency in Zn can lead to a range of health issues, including compromised immunity, growth retardation, appetite abnormalities, and reproductive dysfunction.^[^ ^26^ ^]^ Thus, Zn‐based metal barrier membranes ‌are physiologically compatible‌.

Before clinical applications, the biocompatibility of GBR membranes must be prioritized.‌ Therefore, existing preclinical research predominantly evaluates Zn‐based alloys from two critical perspectives: ‌cytocompatibility‌ and ‌hemocompatibility‌. During degradation in bodily fluids, Zn and its alloys primarily release Zn^2+^ and induce localized ‌pH elevation‌, with ion release levels directly affecting biocompatibility. Generally, current research typically uses the ‌IC_50_ (50% inhibitory concentration)‌ as the benchmark for cytotoxicity. A previous study has indicated that the ‌IC_50_ values of Zn^2+^ for ‌murine osteoblastic cells (MC3T3‐E1 cells)‌ and ‌murine fibroblasts (L929)‌ are ‌90.0‌ and ‌92.8 µm‌, respectively.^[^ ^27^ ^]^ Moreover, ‌Ma et al.‌ recently demonstrated that low Zn^2+^ concentrations (<60 µm) enhance ‌cell viability, proliferation, spreading, and migration‌, while higher concentrations (>140 µm) have inhibitory effects.^[^ ^28^ ^]^ Additionally, Yang et al. systematically investigated the effects of pure Zn and a series of binary Zn‐based alloys on the viability of MC3T3‐E1 cells and human umbilical vein endothelial cells (HUVECs),^[^ ^11^ ^]^ as shown in Figure 3b.‌ Experimental results showed that most Zn‐based alloys exhibit mild cytotoxicity toward osteoblastic cells but maintain favorable cytocompatibility with endothelial cells. Microalloying‌ with pro‐osteogenic elements (e.g., ‌Mg‌, ‌Ca‌, and ‌Li‌) further enhances cellular activity in Zn‐based alloys, suggesting a tunable biocompatibility profile. Furthermore, preliminary cell adhesion assays revealed that preosteoblasts, endothelial cells, and macrophages (stimulated with Lipopolysaccharide (LPS)) adhered to the pure Zn surface but exhibited predominantly rounded morphologies with limited spreading (Figure 3c), likely due to localized Zn^2+^ overconcentration.^[^ ^29^ ^]^ In contrast, human gingival fibroblasts (HGF) demonstrated significantly superior adhesion on the Zn substrate, which is advantageous for promoting epithelial soft tissue regeneration and enhancing wound‐healing efficacy‌.^[^ ^30^ ^]^ The distinct adhesion morphologies exhibited by different cell types on Zn surfaces primarily arise from their varying tolerance thresholds to Zn^2+^, which is a phenomenon well‐documented in previous studies.^[^ ^28^, ^29^, ^31^ ^]^ Moreover, considering the need for barrier membranes to preserve bone defect space throughout the healing cycle‌, a comprehensive assessment of the biocompatibility of Zn‐based alloy degradation products is essential. Previous studies confirm that primary corrosion byproducts, such as zinc oxide (ZnO), zinc phosphate, and zinc carbonate, demonstrate excellent biocompatibility‌,^[^ ^32^ ^]^ highlighting their potential for clinical use in GBR procedures.

In terms of blood compatibility, preliminary studies show that Zn and Zn alloys exhibit superior antiplatelet adhesion properties and lower hemolysis rates‌.^[^ ^29^, ^33^ ^]^ As illustrated in Figure 3d, platelets adhered to Zn surfaces appear uniformly dispersed with minimal activation.^[^ ^34^ ^]^ Furthermore, compared to the medical‐grade WE43 alloy (hemolysis rate ≈40%), Zn‐based metals display significantly lower hemolysis rates (<5%), which is below the ASTM F756‐08 threshold for nonhemolytic materials‌. Yin et al. also revealed that Zn alloys significantly prolong prothrombin time (PT) and activated partial thromboplastin time (APTT) in a dose‐dependent manner, suggesting intrinsic anticoagulant properties by modulating coagulation cascade dynamics‌.^[^ ^33^ ^]^ To investigate the blood compatibility of Zn‐based metals under dynamic flow conditions, Li et al. employed a Chandler‐Loop system to evaluate Zn‐4Cu and Zn‐0.5Cu‐0.2Fe alloy systems (Figure 3e). The data demonstrated that neither alloy induced hemolysis, leukocyte depletion, or platelet aggregation under cyclic shear stress‌.^[^ ^35^ ^]^ This comprehensive analysis supports the superior hemocompatibility of Zn and its alloys, as evidenced by the absence of adverse hematological responses in physiologically relevant flow environments‌.

### 3.2 Biodegradability and Moderate Degradation Rate

**Figure 4:**
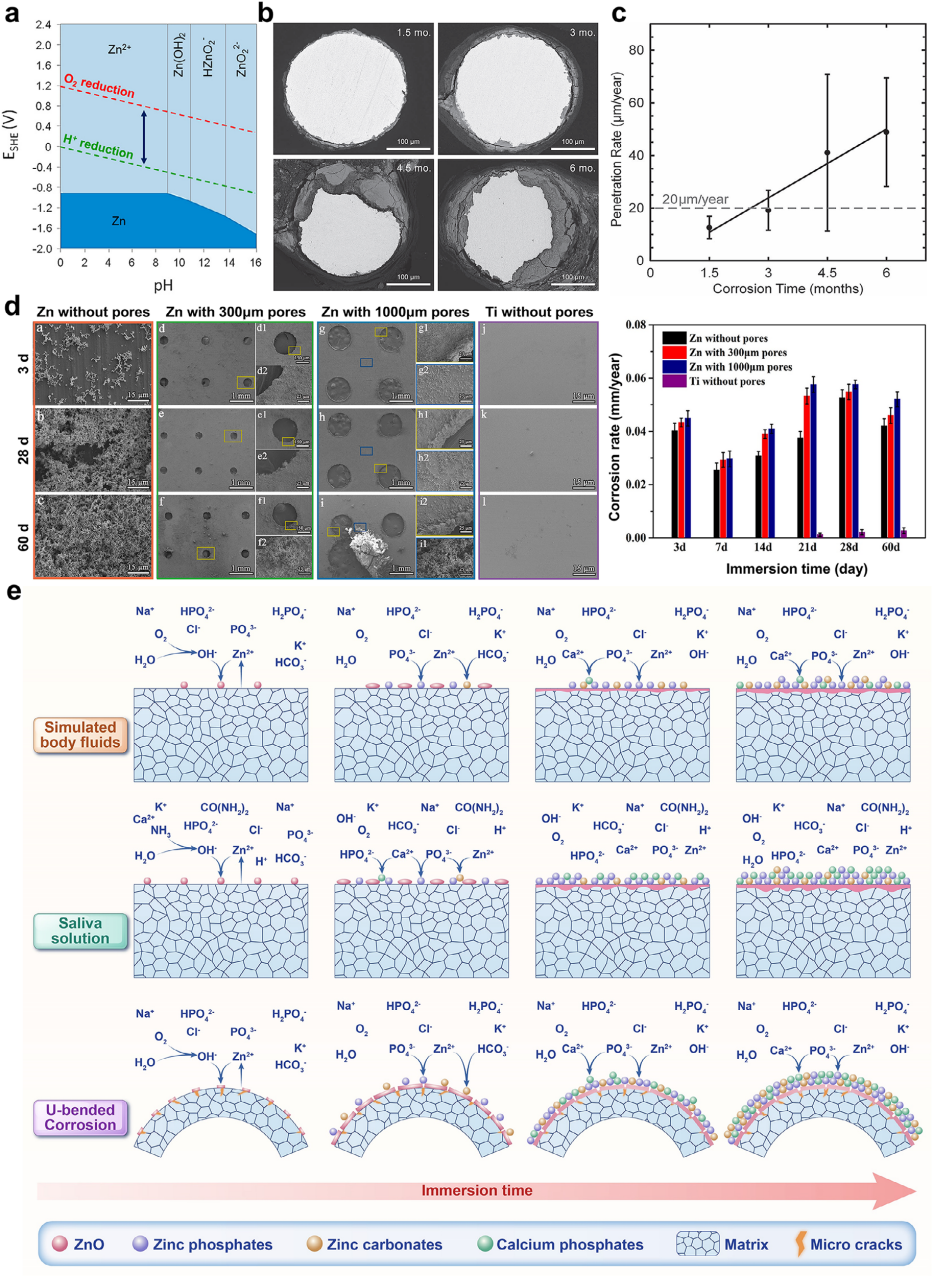
The biodegradation behavior of Zn‐based barrier membrane. a) Pourbaix diagram of Zn (the blue arrow denotes the biological standard reduction potential range at pH 7.4: ‌≈820 to ≈670 mV). Reproduced with permission.^[^ ^49^ ^]^ Copyright 2016, Elsevier. b) The morphological changes in cross‐sectional degradation behavior and c) degradation rates at different time intervals of pure Zn wires implanted in vivo over a 6‐month period. Reproduced with permission.^[^ ^44^ ^]^ Copyright 2013, Wiley‐VCH. d) The surface morphology images and corresponding degradation rates of pure Zn membranes with varying pore sizes (without, 300 and 1000 µm) and a thickness of 30 µm after 60‐day immersion in Hank's solution (Ti membrane without pore was set as control group). Reproduced with permission.^[^ ^18a^ ^]^ Copyright 2020, Elsevier. e) Comparative diagram of degradation mechanisms of pure Zn‌ under simulated body fluid (stress‐free)‌, simulated oral saliva (stress‐free), and simulated body fluid (U‐bending strain)‌ conditions. The schematic configuration of the mechanism diagram derives from results established in our team's prior publications‌.^[^ ^18^, ^29^ ^]^

Zn‐based biodegradable metals are known for their prominent characteristic of biodegradability, allowing them to gradually degrade after completing bone repair in bone defect areas. This process enables the material to be absorbed by human tissues or excreted from the body, effectively avoiding secondary surgery and alleviating patient discomfort‌.^[^ ^37^ ^]^ The primary function of the GBR membrane is to isolate bone defect regions from external soft tissues, with degradation mainly occurring through interactions with physiological fluid environments‌.^[^ ^38^ ^]^ Actually, research on the degradation behavior of Zn‐based metal implants is extensive. In physiological environments, the degradation process involves anodic oxidation (anodic reaction) and the oxygen reduction reaction (cathodic reaction), leading to formation of metal oxides or hydroxides as demonstrated in Equations (1)–(4)‌.^[^ ^39^ ^]^ Unlike Mg‐based alloys, Zn does not generate hydrogen gas during degradation‌.‌ The presence of chloride ion (Cl^−^) in body fluids disrupts the equilibrium between the dissolution and formation of corrosion products on the Zn surface, causing the dissolution of Zn(OH)_2_ and ZnO, which further promotes the precipitation of simonkolleite (Equation (5)).^[^ ^40^ ^]^ ‌Additionally, hydrogen phosphate (HPO_4_^2−^) and bicarbonate (HCO_3_^−^) ions in body fluids accelerate the formation of phosphate and carbonate compounds on the material surface (Equations (6)(7))‌.^[^ ^41^ ^]^ Furthermore, the pH of the electrolyte significantly influences the corrosion behavior of Zn.‌ As depicted in the Pourbaix diagram of Zn (**Figure** **4**a), Zn exhibits higher corrosion rates under both low‐pH (dissolving as Zn^2+^ in acidic solutions) and high‐pH conditions (forming ZnO_2_^2−^ in alkaline environments), while corrosion rates are minimized in neutral conditions‌.^[^ ^42^ ^]^ ‌This supports the notion that Zn undergoes gradual degradation under physiological conditions (pH ≈7.4), making it suitable for fabricating biodegradable GBR membranes‌.

(1) $$\mathrm{Zn}\;\to Zn^{2+}+2e^{-}$$

(2) $$O_{2}+H_{2}O+4e^{-}\;\to 4OH^{-}$$

(3) $$Zn^{2+}+2OH^{-}\;\to \mathrm{Zn}{\left(\mathrm{OH}\right)}_{2}$$

(4) $$\mathrm{Zn}{\left(\mathrm{OH}\right)}_{2}\;\to \mathrm{ZnO}+H_{2}O$$

(5) $$5\mathrm{Zn}{\left(\mathrm{OH}\right)}_{2}+2Cl^{-}+H_{2}O\;\to Zn_{5}{\left(\mathrm{OH}\right)}_{8}Cl_{2}\cdot H_{2}O+2OH^{-}$$

(6) $$3Zn^{2+}+2{\mathrm{HPO}}_{4}^{2-}+2OH^{-}+2H_{2}O\;\to Zn_{3}{\left(PO_{4}\right)}_{2}\cdot 4\;H_{2}O$$

(7) $$5Zn^{2+}+2{\mathrm{HCO}}_{3}^{-}+8OH^{-}\;\to Zn_{5}{\left(CO_{3}\right)}_{2}{\left(\mathrm{OH}\right)}_{6}+2H_{2}O$$

Typically, an ideal GBR membrane generally requires mechanical integrity for ≈16–24 weeks‌.‌^[^ ^3b^ ^]^ This means biodegradable barrier materials must degrade at a rate that aligns with the bone regeneration cycle of alveolar defects‌. ‌Zn, with a standard corrosion potential of −0.76 V, demonstrates moderate degradation kinetics due to its electrochemical position between Mg (−2.37 V) and Fe (−0.44 V)‌.^[^ ^43^ ^]^ Yang et al. systematically investigated the in vitro degradation kinetics of pure Zn and binary Zn alloys, revealing degradation rates of 14–30 µm year^−1^ for Zn‐based alloys, corresponding with the physiological bone remodeling timeline‌.^[^ ^11^ ^]^ Bowen et al. recently investigated the in vivo degradation of pure Zn wire using a Sprague–Dawley rat abdominal aorta model‌, finding that the structural integrity was maintained for at least 4 months (Figure 4b). Subsequent corrosion kinetics exhibited phase acceleration, followed by a transition to accelerated corrosion kinetics‒a critical mechanism ensuring implant degradation synchronization with physiological remodeling phases (Figure 4c)‌.^[^ ^44^ ^]^ Postdegradation analyses at 4.5 and 6 months in vivo revealed that the corrosion products were dense and adherent, helping synchronize implant degradation with bone tissue regeneration. Building on these findings‌, our research team recently fabricated Zn‐based barrier membranes with varying pore diameters (0, 300, and 1000 µm, with pure Ti membranes as the control group). Systematic analysis of in vitro degradation rates revealed that Zn membranes with 300 µm pore configurations demonstrated optimal degradation kinetics, while the 1000 µm variant structurally collapsed after 60 days of exposure‌ to corrosion.^[^ ^18a^ ^]^ Notably, the thickness of Zn membranes significantly affects material degradation kinetics. A previous in vivo study demonstrated that pure Zn membrane (10 µm thickness) remained observable within murine lateral femoral condyles after 3‐month degradation, but was completely resorbed within 6 months‌.^[^ ^45^ ^]^ This temporal degradation profile highlights the feasibility of controlled degradation rates and sustained spatial maintenance in bone defect regions by optimizing Zn‐based membrane thickness‌.

As a GBR membrane, the degradation behavior must account for clinical scenarios‌. Postimplantation, insufficient tissue coverage caused by compromised wound closure or wound dehiscence frequently expose the membrane to the oral environment‌.^[^ ^46^ ^]^ Clinical studies report Ti mesh exposure rates ranging from 20% to 30%, with some cases reaching up to 66%.^[^ ^12b^ ^]^ The oral cavity environment, compared to physiological fluids, is more acidic, which enhances its corrosive potential and accelerates degradation. In a previous study, Zhang et al. compared the degradation behaviors of pure Zn and Zn‐Cu‐Fe alloys in artificial saliva (intraoral environment) and α‐MEM (submucosa environment)‌.^[^ ^47^ ^]^ The results demonstrated that Zn‐based metals degraded faster and exhibited more localized corrosion in oral‐mimetic solutions, as shown in Figure 4e‌. Additionally, to address clinical diverse alveolar bone defect scenarios, intraoperative bending or shaping of barrier membranes is often required‌. This mechanical manipulation may induce stress concentration and stress corrosion cracking at regions of maximal deformation‌. Although Zn exhibits superior stress corrosion resistance‌, prior studies demonstrate that stress concentrations (including tensile, compressive, and bending stresses) can still accelerate degradation and the loss of mechanical integrity in Zn‐based implants‌.^[^ ^18^, ^48^ ^]^ The primary function of barrier membranes is to provide stable mechanical support and spatial stability to bone defect regions‌.^[^ ^2^ ^]^ Therefore, the mechanical degradation caused by stress corrosion behavior requires systematic evaluation to mitigate its impact on membrane's structural integrity‌. Recently, Chen et al. investigated the impact of bending deformation (ϕ 32 mm) on the degradation behaviors and mechanical integrity of pure Zn barrier membranes‌.^[^ ^18b^ ^]^ Their findings revealed that such deformation induces the oxide layer fracture and initiates microcracks on membrane surfaces (Figure 4e), resulting in degradation rates that accelerate by 27.33%–52.77 % across pore sizes ranging from 300 to 1000 µm‌. Importantly, after 45 days of immersion, the membranes retained sufficient mechanical performance to satisfy clinical barrier membrane criteria‌. The study also highlighted that both thickness and pore size critically influence Zn membrane degradation. Specially, the 1000 µm membrane exhibited the highest rate, while the 0 and 300 µm membranes showed similar rates of degradation.^[^ ^18a^ ^]^ Additionally, pure Zn membranes demonstrated lower strength retention (48.10%–68.03%) compared to Zn strips (83.17%), likely due to their increased susceptibility to mechanical failure from reduced thickness.^[^ ^18b^ ^]^ In conclusion, Zn‐based metals fulfill the requirements for degradable GBR membranes owing to their inherent biodegradability and optimal degradation kinetics‌.

### 3.3 Sufficient Mechanical Adaptability

**Figure 5:**
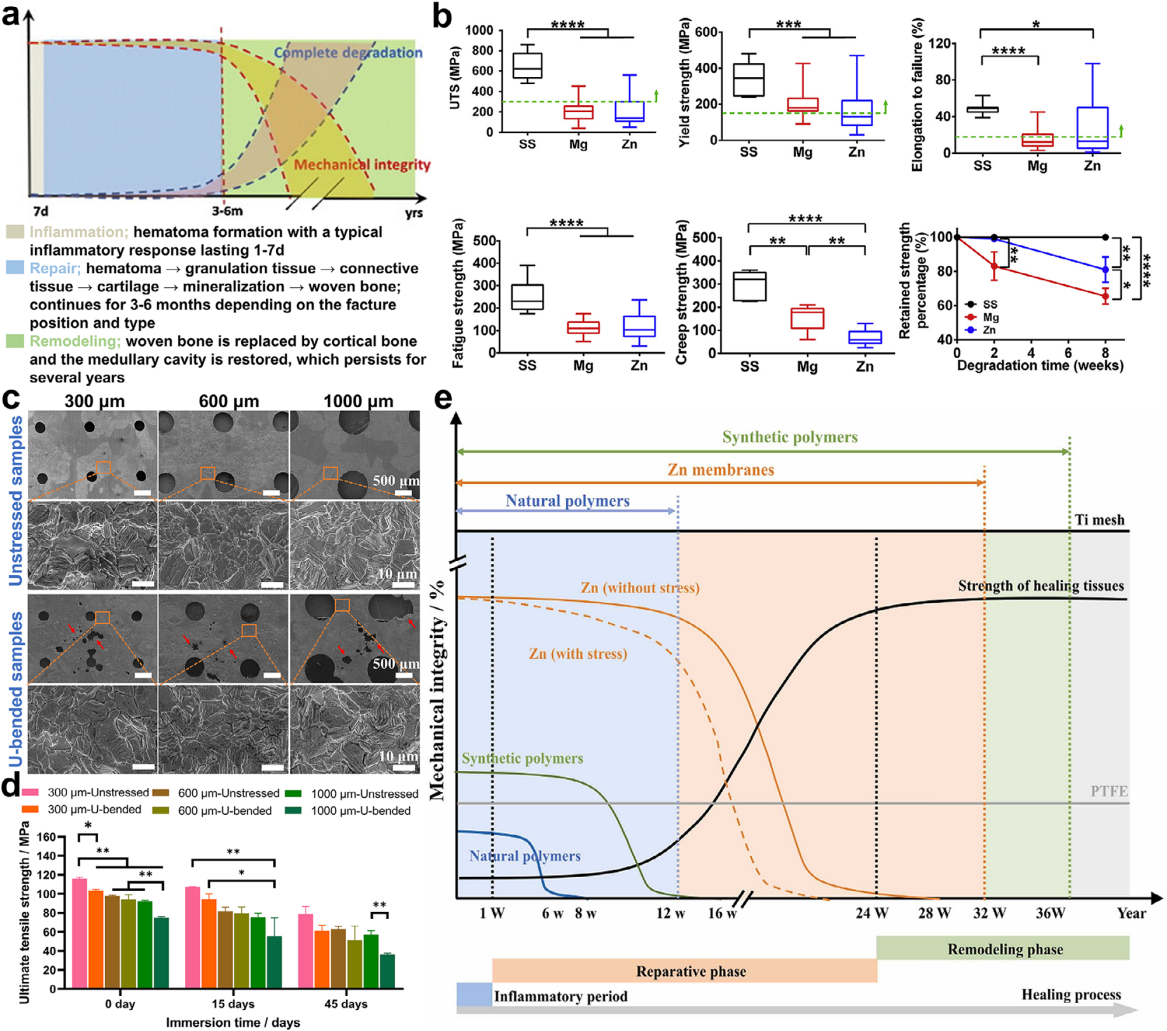
Evaluation of the mechanical properties of Zn‐based barrier membranes. a) Schematic diagram illustrating the relationship between the mechanical integrity of degradable implants and their degradation behavior during the in vivo bone repair process. Reproduced with permission.^[^ ^8a^ ^]^ Copyright 2014, Elsevier. b) The mechanical properties of stainless steel (SS), Mg‐based, and Zn‐based metallic materials (ultimate tensile strength (UTS), yield strength, elongation to failure, fatigue strength, creep strength, and retained strength percentage as a function of degradation time). Reproduced with permission.^[^ ^34^ ^]^ Copyright 2019, Elsevier. c) Corrosion morphology images of pure Zn membranes (with pore sizes of 300, 600, and 1000 µm) at the maximum bending deformation regions, following 45 days of immersion in PBS solution, for nonstressed and postbending deformation conditions. d) Variation trend of tensile strength in Zn‐based barrier membranes at different time intervals. e) Schematic diagram illustrating the correlation between mechanical integrity and degradation time for various GBR membrane materials. Reproduced with permission.^[^ ^18b^ ^]^ Copyright 2024, Elsevier.

An ideal barrier membrane must exhibit sufficient mechanical adaptability. The mechanical compatibility of the barrier membrane primarily encompasses two aspects‌. First, compared to other degradable GBR membrane materials, it must exhibit superior mechanical properties (such as tensile strength and flexural strength) to effectively isolate the bone defect area from epithelial soft tissues‌. Second, the barrier membrane must retain excellent mechanical integrity throughout its degradation process.^[^ ^50^ ^]^ This ensures that it continues to meet mechanical performance requirements in the bone defect gap until the entire alveolar bone repair cycle is completed‌. Bone tissue repair generally progresses through three primary phases: the ‌early inflammatory phase‌ (1–7 days), the ‌bone repair phase‌ (2–3 weeks), and the ‌bone remodeling phase‌ (3–6 months), as illustrated in **Figure** **5**a.^[^ ^8a^ ^]^ Therefore, the degradation kinetics of ‌biodegradable implants‌ during bone repair must ensure ‌sustained mechanical integrity‌ throughout the critical ‌3–6 month period‌‌.^[^ ^51^ ^]^ Recent studies by Chen et al. systematically compared the mechanical properties of medical‐grade biodegradable polymer barrier membranes, demonstrating maximum tensile strength and elongation values of ‌55 MPa‌ and ‌20%‌, respectively‌.^[^ ^4a^ ^]^ In contrast, Zn‐based biodegradable metals‌ exhibit superior mechanical performance, including tensile strength, elongation, fatigue resistance, and creep resistance (Figure 5b‌).^[^ ^34^ ^]^ These properties ensure ‌sustained mechanical integrity‌ for bone defect regeneration‌. For instance, a study by Guo et al. have demonstrated that pure Zn membrane maintains structural integrity without rupture for up to 10 weeks postimplantation in in vivo rat calvarial defect models,^[^ ^18a^ ^]^ confirming its sufficient mechanical durability under physiological loading conditions‌. However, complex bone augmentation procedures in clinical surgery may exceed the mechanical capacity of pure Zn barrier membranes for sustained space maintenance when compared to pure Ti membrane. To address this limitation, our team previously developed binary and ternary Zn‐based alloys through microalloying with essential trace elements (e.g., Li, Sr, Mg). This resulted in Zn‐Li‐Sr alloys with a tensile strength of ‌524.3 MPa‌ and elongation of ‌25%‌.^[^ ^11^, ^52^ ^]^ These values exceed those of conventional pure Ti implants (≈500 MPa), demonstrating superior mechanical stability for load‐bearing defect repair and osteogenic space preservation‌.

As previously discussed, intraoperative bending and plastic deformation of barrier membranes during clinical procedures can initiate SCC‌. While Zn‐based metals exhibit lower SCC susceptibility than other biodegradable metals, studies have shown that SCC still compromises their mechanical integrity under physiological stress conditions‌. One study demonstrated that a ‌25% reduction in time to failure‌ during SCC testing in modified simulated body fluid (m‐SBF) and whole blood environments‌.^[^ ^53^ ^]^ In addition, our prior study demonstrated a ‌19.6% reduction in tensile strength‌ in 10% prestrained pure Zn wires (φ 0.5 mm) after 20 days of immersion in Dulbecco's modified eagle medium (DMEM)‌.^[^ ^48a^ ^]^ These findings highlight the significant impact of the interaction between applied mechanical loads/strains and corrosive degradation on the mechanical integrity of barrier membranes‌. Such degradation mechanisms may critically affect long‐term maintenance of bone defect spaces and the efficacy of osseous regeneration‌. To address this challenge, our research group previously simulated the synergistic effects of U‐bending deformation (with/without prestrain) and immersion in phosphate‐buffered saline (PBS) for 45 days on the mechanical integrity of pure Zn strips and barrier membranes.^[^ ^18b^ ^]^ The results revealed that prestrain accelerates material degradation, with mechanical integrity loss rates reaching ‌54.14%–64.24%‌ for pure Zn barrier membranes, which are significantly lower than those of polymer‐based counterparts‌. The primary mechanism is attributed to stress concentration effects caused by prestrain at the membrane's maximum deflection zones, which triggers rupture of surface protective layers and microcrack nucleation (Figure 5c), thus accelerating localized corrosion. Nevertheless, Zn‐based barrier membranes (300 and 600 µm) retain compliance with mechanical performance criteria for barrier membranes even after 45‐day corrosion exposure (Figure 5d). Consequently, Zn‐based metallic barrier membranes exhibit optimal mechanical adaptability throughout the alveolar bone regeneration cycle (Figure 5e). They degrade after guided bone repair and are either metabolically absorbed or excreted by the body.

### 3.4 Guided Bone Regeneration Activity

**Figure 6:**
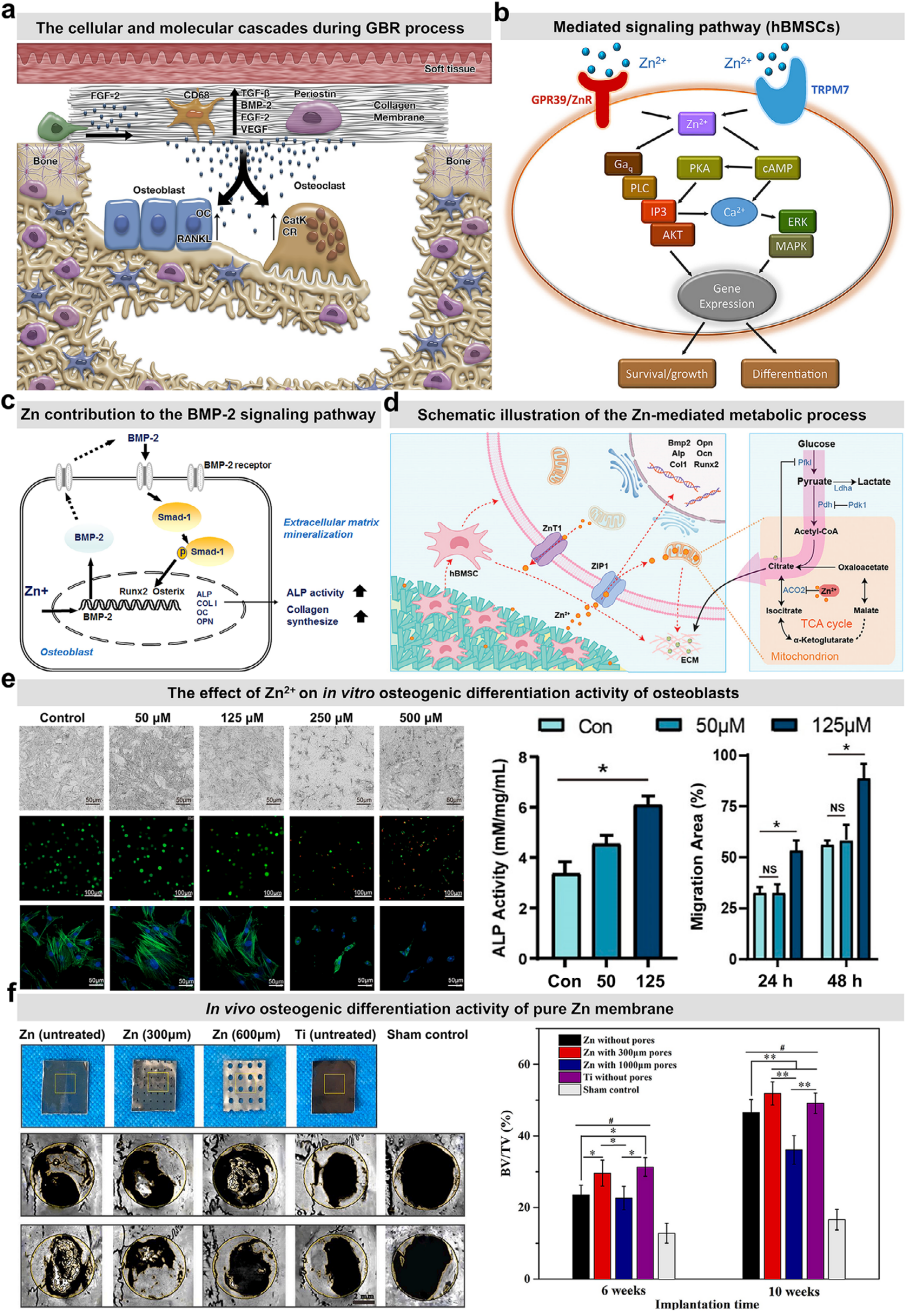
Cellular responses to Zn‐based barrier membranes, underlying osteogenic mechanisms, and in vitro/in vivo bone regenerative activities. a) Cellular and molecular mechanisms underlying guided bone regeneration therapy. Reproduced with permission.^[^ ^54^ ^]^ Copyright 2017, John Wiley & Sons Ltd. b) Zn^2+^ released from implants engages GPR39/ZnR‐TRPM7 receptors in hBMSCs, sequentially activating cAMP‐PKA/Gaq‐PLC‐IP3 pathways to drive Ca^2+^‐mediated MAPK/AKT signaling and transcriptional regulation of cellular differentiation and proliferation. Reproduced with permission.^[^ ^62^ ^]^ Copyright 2017, American Chemical Society. c) Zn‐mediated modulation of BMP‐2 signaling in osteoblast regulation. Reproduced with permission.^[^ ^65^ ^]^ Copyright 2018, The Korean Nutrition Society. d) Zn^2+^ promotes osteogenic differentiation and ECM mineralization in hBMSCs by reprogramming citrate metabolism (enhanced synthesis/reduced catabolism). Reproduced with permission.^[^ ^70^ ^]^ Copyright 2024, Wiley‐VCH. e) The effects of varying Zn^2+^ concentrations on the cellular morphology (optical images, live/dead staining, and fluorescence observation results), ALP activity, and migration capacity of human bone marrow mesenchymal stem cells (hBMSCs).‌ ‌Reproduced with permission.^[^ ^71^ ^]^ Copyright 2024, American Chemical Society. f) The bone repair activity outcomes of pure Zn barrier membranes with varying pore sizes in a rat calvaria bone defect repair model. Reproduced with permission.^[^ ^18a^ ^]^ Copyright 2020, Elsevier.

The most critical function of a barrier membrane is to guide bone repair within the defect area, which necessitates that ideal barrier membrane materials possess superior osteogenic activity.‌ As shown in **Figure** **6**a, the schematic diagram comprehensively depicts the spatial configuration of the ideal GBR membrane and its associated cellular/molecular mechanisms‌.^[^ ^54^ ^]^ Monocytes, macrophages, and osteoprogenitors initially migrate from peripheral tissues into the membrane microenvironment, where they subsequently express and secrete key osteogenic mediators, including BMP‐2, TGF‐β, VEGF, and FGF‐2, to coordinate both bone formation and remodeling processes‌. Simultaneously, the GBR membrane dynamically modulates pro‐osteogenic markers (e.g., RANKL) and osteoclastic indicators (e.g., cathepsin K, calcitonin receptor) at the defect site through integrated cellular signaling pathways and biomolecular interactions‌. Therefore, the optimal barrier membrane must integrate osteoinductive capabilities to drive osteoblastic differentiation and mineralization, thus facilitating expedited bone regeneration‌. For Zn‐based implants, they primarily mediate bone microenvironment regeneration via endogenously degraded Zn^2+^, which coordinates osteoblast‐osteoclast equilibrium and activates Zn‐dependent transcriptional pathways‌. During bone healing, Zn^2+^ degradation byproducts engage in complex interactions with key skeletal cell populations (i.e., osteoblasts, osteoclasts, endothelial cells, and immune cells) that collectively govern host responses to Zn‐based implants through coordinated regulation of osteogenic differentiation‌, angiogenic signaling‌, and immunomodulatory pathways‌.^[^ ^55^ ^]^ To date, preliminary in vitro and in vivo investigations (i.e., rabbit femoral shaft fracture model, rat femoral condyle defect repair model, beagle canine mandibular facture model) have confirmed that Zn‐based metals/composites maintain favorable biocompatibility profiles during prolonged in vivo degradation processes.^[^ ^56^ ^]^ Hence, a comprehensive analysis of Zn^2+^ spatiotemporal dynamics enables precise prediction of Zn‐based implants' clinical efficacy, as ionic distribution patterns govern osteogenic activity and immunomodulatory responses. Generally, Zn^2+^ generally plays a number of roles in bone regeneration and repair, such as enhancing osteoblastic bone formation, inhibiting osteoclastic bone resorption, and promoting osteogenic differentiation of mesenchymal stem cells (MSCs).^[^ ^57^ ^]^ Additionally, Zn^2+^ may promote immunomodulation and angiogenesis, which would result in the production of new bone.^[^ ^58^ ^]^

In terms of Zn‐mediated osteogenic differentiation, previous studies indicate that Zn^2+^ enhances osteogenic differentiation by promoting MSCs proliferation, alkaline phosphatase (ALP) activity, and calcium deposition.^[^ ^59^ ^]^ In the early stage of osteogenic differentiation, extracellular Zn^2+^ upregulates the expression of MSC‐specific genes including ALP, runt‐related transcription factor 2 (Runx‐2), and type 1 collagen,^[^ ^60^ ^]^ while Zn supplementation during middle‐to‐late stages markedly enhances MSC expression of late osteogenic markers such as osteocalcin and osteopontin.^[^ ^61^ ^]^ In general, the molecular mechanism underlying Zn‐mediated regulation primarily involves activation of the mitogen‐activated protein kinase (MAPK) pathway, protein kinase B (AKT) pathway, protein kinase A (PKA) pathway, and transforming growth factor β (TGF‐β) signaling pathway.‌^[^ ^24a^ ^]^ Recently, Zhu et al. established Zn^2+^‐mediated signaling mechanisms in hBMSCs: TRPM7 and GPR39 were identified as primary Zn^2+^ influx channels‌.^[^ ^62^ ^]^ Intracellular Zn^2+^ activates both cAMP‐PKA signaling and Ca^2+^‐mediated MAPK cascades, while concomitantly stimulating the Gαq‐PLC‐AKT axis (Figure 6b). These coordinated pathways drive osteogenic differentiation through transcriptional reprogramming, ECM mineralization, and enhanced cellular viability‌. Park et al. also demonstrated that Zn^2+^ dose‐dependently increase intracellular cAMP levels, enhance PKA activity through the cAMP‐PKA‐CREB axis, and promote osteogenic differentiation in human BMSCs via Runx‐2 activation in downstream signaling pathways.‌^[^ ^63^ ^]^ Additionally, the activation of TGF‐β/BMP signaling is also implicated in Zn‐induced osteogenic differentiation of MSCs.^[^ ^64^ ^]^ Moreover, Zn deficiency in osteoblasts induces Smad‐1 activation and Runx‐2 downregulation through BMP‐2 signaling, suppressing osteoblast differentiation, whereas elevated Zn levels enhance both Runx‐2 expression and Smad‐1 activation (Figure 6c), demonstrating Zn's promotion of osteogenesis via canonical BMP‐2 signaling.‌^[^ ^65^ ^]^ Beyond that, Zn not only encourages osteogenic differentiation but also regulates osteoclastogenesis. Zn‐mediated inhibition of osteoclastogenesis primarily involves the NFATc1 and NF‐κB signaling pathways,^[^ ^66^ ^]^ with experimental evidence demonstrating that Zn^2+^ suppresses calcineurin activity during early osteoclastogenesis and inhibits calcium influx‐mediated oscillations by blocking extracellular calcium entry at mid‐late differentiation stages through the Ca^2+^‐calcineurin‐NFATc1 axis.^[^ ^67^ ^]^ Hie and Tsukamoto found that Zn administration inhibits osteoclastogenesis through downregulating NF‐κB receptor (RANK) levels via suppression of reactive oxygen species (ROS) production and extracellular signal‐regulated kinase (ERK) activity.^[^ ^68^ ^]^ Zn^2+^ can also orchestrate metabolic‐osteoclastic crosstalk through RANK/RANKL/OPG pathway regulation, mediating bidirectional signaling between osteoblast activity and osteoclastic differentiation processes.^[^ ^69^ ^]^ However, the Zn‐mediated signaling crosstalk between osteogenic and osteoclastogenic pathways necessitates further mechanistic interrogation to elucidate its regulatory dynamics‌. Furthermore, Zhao et al. discovered that Zn^2+^ could regulate osteogenic differentiation in hBMSCs by activating ZIP/ZnT transporters to enhance intracellular Zn^2+^ levels, which subsequently reprogram cellular metabolism through citrate accumulation via suppressed TCA cycle enzymes (PFK1, LDHA) and mitochondrial aconitase inhibition, ultimately promoting ECM mineralization.^[^ ^70^ ^]^ This Zn‐mediated metabolic shift increases acetyl‐CoA flux for citrate synthesis while reducing ATP production, triggering compensatory glucose utilization and mitochondrial elongation to meet biosynthetic demands during bone formation (Figure 6d). The coordinated effects of Zn^2+^ on both gene expression and mitochondrial morphology (evidenced by tubular transformation) demonstrate its dual regulatory capacity in osteogenesis and biomineralization processes.

Notably, Zn^2+^ actually exhibit a dose‐dependent therapeutic profile on the osteogenic differentiation of periosteal cells, where lower concentrations of extracellular Zn^2+^ enhance the osteogenic differentiation of periosteal cells, while higher concentrations of Zn^2+^ exert an inhibitory effect.‌^[^ ^24a^ ^]^ Recent studies by Liu et al. revealed Zn^2+^ concentration‐dependent dual regulatory effects on hBMSCs: physiological levels (≤125 µm) enhance cellular viability, proliferation, migratory capacity, and osteogenic differentiation potential, whereas elevated concentrations (>250 µm) suppress osteoblast functionality and induce apoptosis through disrupted zinc homeostasis, as illustrated in Figure 6e.‌^[^ ^71^ ^]^ Li et al. also demonstrated concentration‐dependent effects of Zn extracts on periosteal stem cells, with low‐concentration formulations enhancing osteogenic differentiation capacity while high‐concentration counterparts suppressed calcium deposition processes.‌^[^ ^32b^ ^]^ A prior research‌ demonstrated dose‐dependent enhancement of MC3T3‐E1 preosteoblast proliferation, peaking at 50 µm Zn^2+^ with significant inhibition observed beyond 130 µm.^[^ ^72^ ^]^ Further, He et al. recently reported that the critical threshold for Zn^2+^ release determining in vitro biocompatibility of Zn‐based biomaterials was established at ≈0.3 mm‌.^[^ ^73^ ^]^ This biphasic response‌ reveals Zn's dual role in bone metabolism: optimal concentrations promote osteogenesis and tissue remodeling, while deviations from the therapeutic window can exhibit cytotoxic potential‌. One molecular mechanism underlying this inhibition involves zinc disrupting crucial signaling pathways and regulatory molecules that coordinate osteogenic differentiation. Notably, it perturbs intracellular signaling balances, including apoptosis‐related pathways like the Wnt/β‐catenin pathway, which is essential for osteoblast differentiation and bone formation.^[^ ^74^ ^]^ To validate in vivo efficacy, we systematically evaluated pure Zn membranes with varying pore sizes (0, 300, and 1000 µm) for cranial defect repair in rat models. After 10 weeks, the 300 µm‐pore Zn membranes outperformed Ti controls in bone regeneration, demonstrating continuous osseointegration, a thicker neocortex, and synchronized remodeling at the bone‐Zn interface. In contrast, 1000 µm‐pore Zn membranes failed due to structural collapse from inadequate mechanical strength, hindering repair (Figure 6f).^[^ ^18a^ ^]^ Collectively, our integrated in vivo and in vitro evidence underscores the significant potential of Zn‐based barrier membranes for guided bone regeneration.

**Figure 7:**
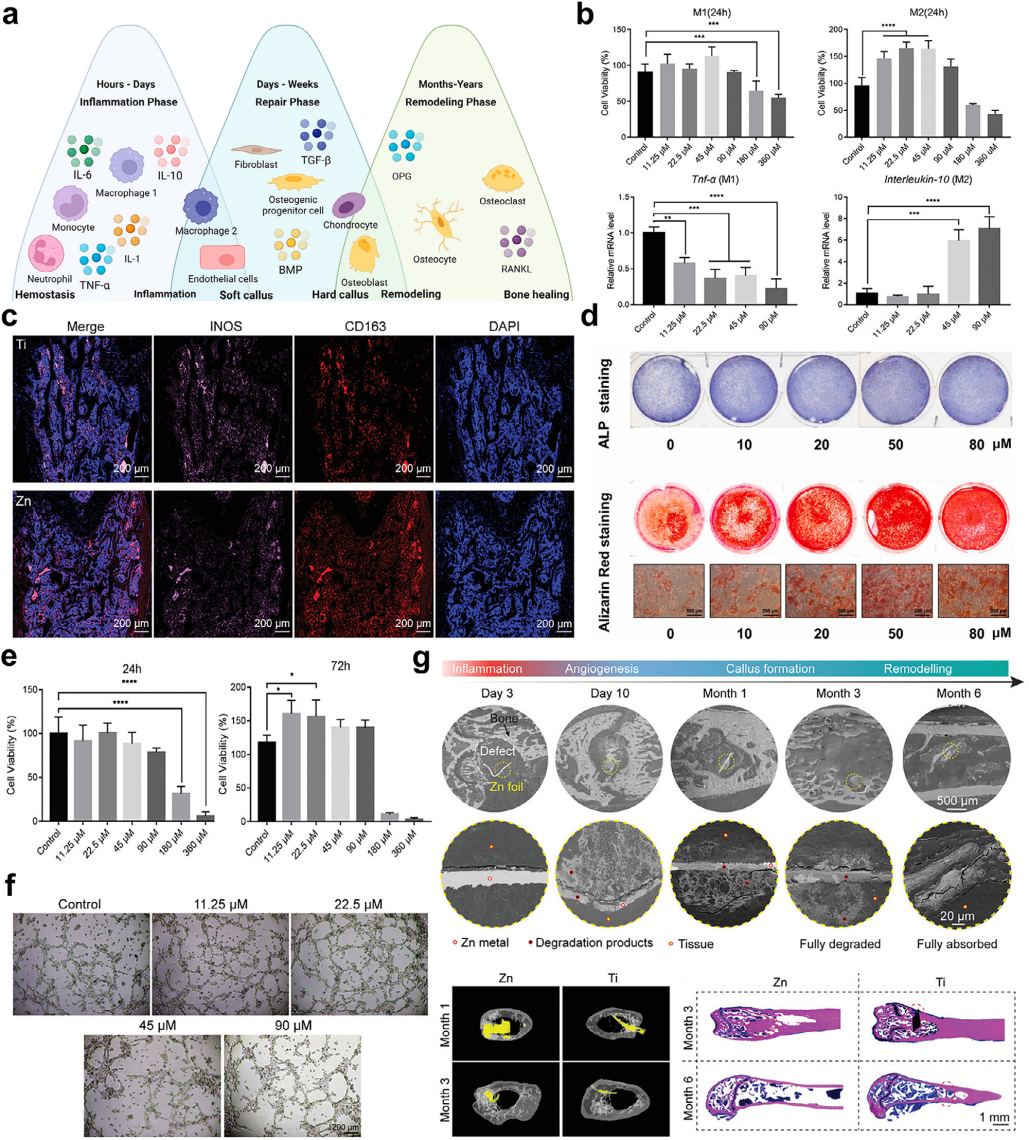
The immunomodulatory and angiogenic properties of Zn‐based barrier membranes. a) The bone regeneration process critically relies on immune modulation (via macrophage polarization and cytokines IL‐10/IL‐4) and angiogenesis, which synergistically orchestrate osteogenesis through hematopoietic cell recruitment, revascularization, and osteoblast/osteoclast coupling mediated by TGF‐β, BMP, and RANKL/OPG signaling. Reproduced with permission.^[^ ^2^ ^]^ Copyright 2023, American Chemical Society. b) The effects of pure Zn at different concentrations on macrophage cellular viability and polarization in vitro. c) Immunofluorescence staining results of macrophages (iNOS, CD163, DAPI) surrounding pure Zn and pure Ti implants in rat femoral condyle defects at 10 days postimplantation. Reproduced with permission.^[^ ^45^ ^]^ Copyright 2023, Wiley‐VCH. d) Staining results of osteogenic differentiation in hBMSCs induced by conditioned medium from macrophage‐zinc ion cocultures at varying concentrations (ALP: Alkaline Phosphatase; ARS: Alizarin Red S). Reproduced with permission.^[^ ^77^ ^]^ Copyright 2020, Elsevier. Effects of Zn^2+^ at varying concentrations on HUVEC e) cellular viability and f) tube formation. g) Biodegradation and bone repair outcomes of pure Zn membranes in murine femoral condyle defects over a 6‐month implantation period (including Micro‐CT and Methylene Blue‐Acid Fuchsin staining results). Reproduced with permission.^[^ ^45^ ^]^ Copyright 2023, Wiley‐VCH.

The bone regeneration process involves coordinated immune modulation and vascularization across inflammatory, repair, and remodeling phases, as shown in **Figure** **7**a. Initially, infiltrating immune cells like macrophages secrete cytokines (IL‐1, TNF‐α) to clear debris and trigger repair, while anti‐inflammatory signals (IL‐10, IL‐4) later shift macrophages to prohealing M2 phenotypes.^[^ ^2^ ^]^ Revascularization and hard callus formation are critically driven by cytokines (TGF‐β, BMP) alongside mesenchymal cell differentiation into osteoblasts and chondrocytes. Finally, RANKL/OPG‐regulated osteoclast‐osteoblast interactions remodel immature bone into mature lamellar bone, emphasizing the dual role of immune cues and vascular repair in achieving functional restoration. Notably, Zn‐based barrier membranes offer a unique benefit, as their primary degradation byproduct, Zn^2+^, exert regulatory effects on both immunomodulation and angiogenesis.^[^ ^75^ ^]^

Thus far, immunomodulatory strategies centered on biomaterials for tissue regeneration have predominantly aimed at modulating macrophage polarization and regulating T lymphocyte homeostasis.^[^ ^76^ ^]^ Experimental data reveal that Zn^2+^ concentrations of ≈11.25–45 µm released from degrading Zn‐based implants selectively stimulate M0/M2 macrophage proliferation without altering M1 population dynamics, as documented in Figure 7b.^[^ ^45^ ^]^ Besides, Zn^2+^ concentrations spanning ≈11.25–90 µm effectively facilitate phenotypic conversion of macrophages from the M0 quiescent state to the immunoregulatory M2 subtype‌. In a recent rat femoral condyle defect model with pure Zn and Ti membranes, Yang et al. revealed significantly elevated CD163 expression (a specific marker for M2‐subtype macrophages) in the Zn group compared with pure Ti counterparts at 10‐day postimplantation, as documented in Figure 7c.^[^ ^45^ ^]^ This in vivo evidence substantiates Zn's capacity to potentiate M2 macrophage‐mediated immunomodulatory effects. To further investigate the impact of Zn‐modulated immune microenvironments on hBMSCs osteogenic differentiation, Ji et al. demonstrated that Zn^2+^‐enriched macrophage‐conditioned medium (≈20–80 µm) established an immunomodulatory microenvironment. This microenvironment significantly enhanced hBMSCs osteogenesis, evidenced by coordinated upregulation of both early‐stage (ALP, Col1, Runx‐2, OSX) and late‐stage (OCN, OPN) osteogenic genes, accompanied by temporally phased functional enhancements: peak ALP activity at ≈20–50 µm concentrations on day 7 and maximal calcium nodule formation at 50–80 µm by day 14 (Figure 7d).^[^ ^77^ ^]^ Implant‐derived Zn^2+^ orchestrates a regenerative cascade through three key mechanisms: 1) selective promotion of M2 macrophage polarization, which establishes an anti‐inflammatory environment; 2) immune cell secretion of osteogenic cytokines, such as BMP‐2 and TGF‐β, that trigger osteoblast differentiation; and 3) direct enhancement of osteoblast activity while suppressing osteoclastogenesis. This dual‐pathway reprogramming increases ALP activity, collagen deposition, and mineral apposition rates. The time‐dependent synergy—transitioning from acute inflammation to chronic remodeling—results in significantly greater bone volume in Zn‐implanted defects, highlighting Zn's unique ability to coordinate immunomodulation with osteogenesis. Moreover, Zn supplementation (50 µm) induces Treg polarization by suppressing Th1 cytokine production (e.g., IFN‐γ) and enhancing TGF‐β1/Smad2/3 signaling, which stabilize forkhead box P3 expression.^[^ ^78^ ^]^ Additionally, Zn^2+^ enhances the immune response of macrophages by modulating the SIRT1/FoxO1 signaling pathway, which regulates glucose metabolism and mitochondrial function.^[^ ^79^ ^]^ Conversely, Zn deficiency impairs macrophage phagocytic capacity, increasing susceptibility to infections.^[^ ^80^ ^]^

On the other hand, the materials of the barrier membrane should promote angiogenesis, thus facilitating blood supply of the bone regeneration area. The interdependence between angiogenesis and osteogenesis is critical, as impaired angiogenesis elevates the risk of nonunions and delays bone repair.^[^ ^81^ ^]^ Notably, studies demonstrate that Zn^2+^ exert proangiogenic effects in a dose‐dependent manner. Our prior studies revealed that low concentrations of Zn^2+^ (below 90 µm) significantly enhance HUVEC proliferation, while concentrations exceeding 90 µm trigger apoptosis and cytotoxicity.^[^ ^45^ ^]^ Sreenivasamurthy et al. reported that Zn^2+^ demonstrates a dual‐phase, dose‐dependent proangiogenic effect, with low concentrations (e.g., 10 µm) promoting endothelial cell proliferation, migration, and tube formation in vitro, while higher concentrations (e.g., 50 µm) enhance in vivo vascularization by upregulating vascular endothelial growth factor (VEGF) and fibroblast growth factor (FGF) signaling pathways.^[^ ^82^ ^]^ As previously noted in mesenchymal stem cells (MSCs), physiological concentrations of Zn (25 µm) activate the Zn‐sensing receptor ZnR/GPR39 through G‐protein coupling, initiating Gαq‐mediated signaling cascades. This activation sequentially engages phospholipase C (PLC), AKT, mitogen‐activated protein kinase (MAPK), phosphoinositide 3‐kinase (PI3K), and extracellular signal‐regulated kinase (ERK)1/2 pathways, collectively regulating essential endothelial cell functions including survival, proliferation, inflammatory responses, and angiogenesis.^[^ ^83^ ^]^ The transcription factor hypoxia‐inducible factor 1 alpha (HIF‐1α) serves as a critical regulator of VEGF expression via NF‐κB pathway activation.^[^ ^84^ ^]^ While HIF‐1α stabilization typically occurs under hypoxic conditions or elevated lactate concentrations, elevated Zn levels demonstrate paradoxical regulatory effects. Specifically, increased Zn^2+^ concentrations suppress HIF‐1α accumulation under hypoxia, thereby inhibiting subsequent VEGF expression.^[^ ^85^ ^]^ Beyond that, Zn^2+^ indirectly modulate angiogenesis by polarizing macrophages toward the M2 anti‐inflammatory phenotype, which secretes proangiogenic factors (e.g., TGF‐β), synergizing with Zn's direct effects to establish a vascular regeneration‐promoting microenvironment.^[^ ^58^, ^86^ ^]^

### 3.5 In Situ Bacteriostatic Activity Against Oral Infection

**Figure 8:**
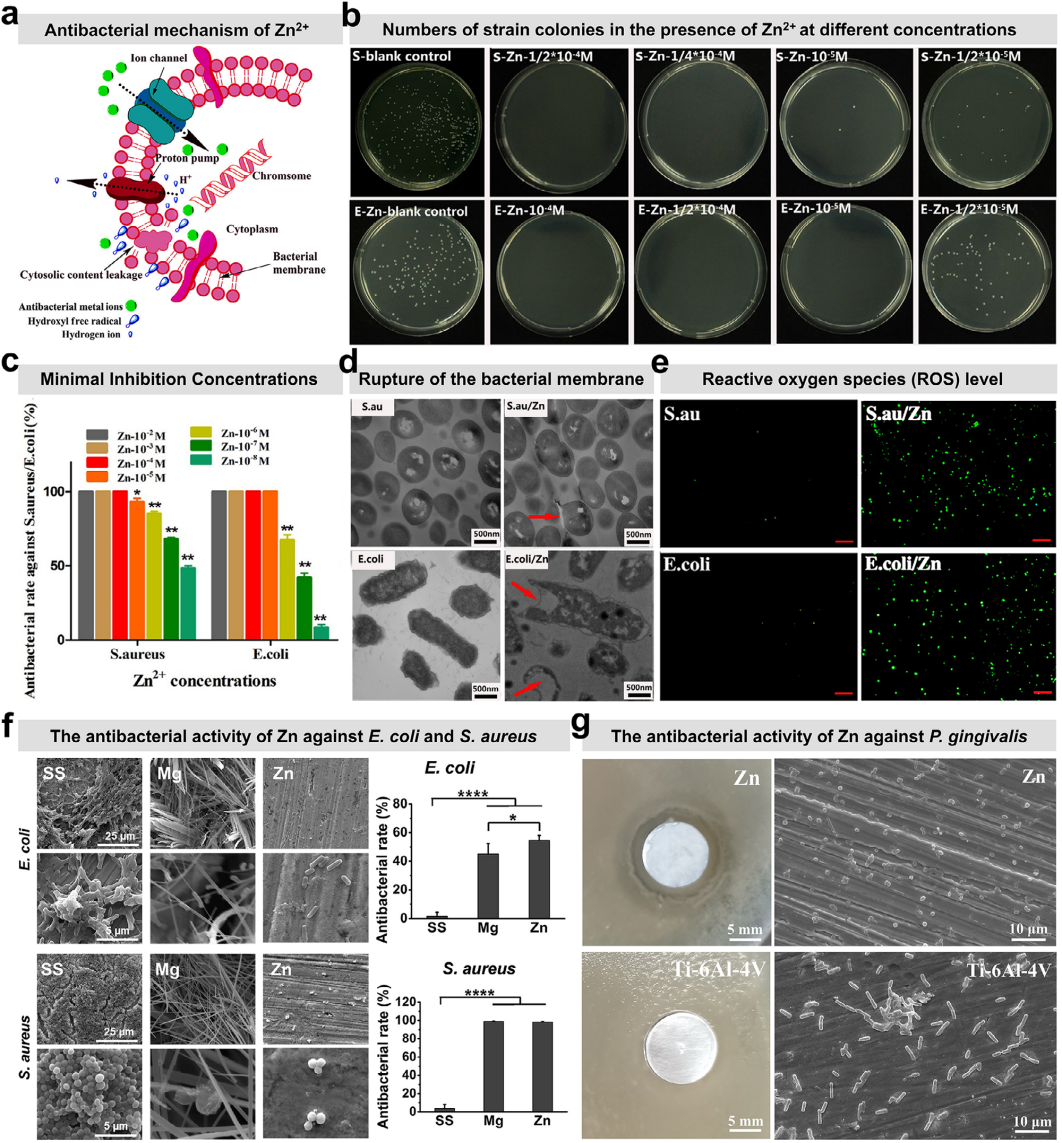
Analysis of antimicrobial activity of Zn‐based barrier membranes. a) Visual schematic outlining the potential antibacterial mechanism of Zn^2+^. b) Colony counts of *S. aureus* and *E. coli* after 24‐h coculture with Zn^2+^ at varying concentrations (initial bacterial concentration: 10^6^ CFU mL^−1^). c) Antibacterial rates of Zn^2+^ at different concentrations against both bacterial strains. d) TEM images of bacterial morphology post‐coculture with Zn^2+^. e) DCF fluorescence images of both bacterial strains treated with/without Zn^2+^ (10 µm). Reproduced with permission.^[^ ^88b^ ^]^ Copyright 2015, American Chemical Society. f) Surface bacterial adhesion morphology and corresponding antibacterial rates of three typical biomedical metals (Stainless steel: SS, WE43 Mg alloy: Mg, pure Zn: Zn) cocultured with *S. aureus* and *E. coli* for 24 h. Reproduced with permission.^[^ ^34^ ^]^ Copyright 2019, Elsevier. g) Inhibition zone results and surface‐adhered bacterial morphology of pure Zn and Ti‐6Al‐4V alloy after 24‐h coculture with *P. gingivalis*. Reproduced with permission.^[^ ^29c^ ^]^ Copyright 2021, The Royal Society of Chemistry.

It is conceivable that inadequate tissue regeneration or wound dehiscence during the postoperative period often leads to barrier membrane exposure to the oral environment. The oral cavity harbors a complex polymicrobial ecosystem comprising diverse aerobic and facultative anaerobic bacteria.^[^ ^87^ ^]^ Contamination by oral microbial communities is a well‐established contributor to implant failure. Therefore, an ideal barrier membrane material should also exhibit robust broad‐spectrum antibacterial activity to mitigate contamination by diverse oral bacterial species. During the in vivo degradation of Zn‐based barrier membranes, a release of Zn^2+^ from the matrix occurs continuously. The Zn^2+^ exhibits broad‐spectrum antibacterial effects, primarily attributed to the following mechanisms (**Figure** **8**a):‌ ‌1) Electrostatic disruption of bacterial membranes‌: Zn^2+^ released from the matrix carries a positive charge, while bacterial surfaces are negatively charged. Coulombic attraction drives Zn^2+^ adhesion to bacterial cell walls, inducing structural damage and subsequent leakage of intracellular components (e.g., lactate dehydrogenase (LDH)).^[^ ^88^ ^]^ 2) Protein denaturation and enzyme inactivation‌: Zn^2+^ binds to intracellular proteins or anionic functional groups, causing protein conformational changes, and deactivation of biosynthetic enzymes. This disrupts bacterial proliferation and growth.^[^ ^89^ ^]^ 3) *Interaction and metabolic interference*‌: Zn^2+^ reacts with bacterial DNA, impairing critical functional systems (e.g., replication and repair) and obstructing metabolic pathways, ultimately leading to bacterial death.^[^ ^90^ ^]^ Consequently, the antibacterial efficacy of Zn‐based membranes is predominantly governed by the release kinetics of Zn^2+^.‌ Ning et al. recently investigated the antibacterial behavior of Zn^2+^ at varying concentrations against *Staphylococcus aureus* (*S. aureus*) and *Escherichia coli* (*E. coli*), as well as the viability trends of mouse fibroblast L929 cells. Experimental results demonstrated that the minimum bactericidal concentration (MBC) and minimum inhibitory concentration (MIC) of Zn^2+^ were 10 µm (Figure 8b) and 0.1 µm (Figure 8c), respectively.^[^ ^88b^ ^]^ Notably, when Zn^2+^ concentrations were below 100 µm, the viability of L929 cells remained above 80%. Additionally, experimental results demonstrated that the presence of Zn^2+^ significantly elevated intracellular reactive oxygen species (ROS) generation in bacteria (Figure 8e), concurrently accelerating membrane disruption and permeability enhancement (Figure 8d). A recent study has further demonstrated that pure Zn exhibits significantly enhanced antimicrobial activity and antibiofilm efficacy against *S. aureus* and *E. coli* compared to Mg‐ and stainless steel‐based implant materials, as illustrated in Figure 8f.^[^ ^34^ ^]^ Moreover, prior comparative studies by our team revealed that pure Zn exhibits superior antibacterial efficacy against *Porphyromonas gingivalis* (*P.gingivalis*, a representative aerobic bacterium in the oral cavity) compared to Ti‐6Al‐4V, with results corroborated in Figure 8g. Furthermore, given the complex polymicrobial nature of the oral environment, Li et al. incorporated trace Fe and Cu into Zn matrix. This alloy design significantly accelerated the release of Zn^2+^, thereby endowing Zn‐Cu‐Fe implants with enhanced antibacterial efficacy against multispecies oral biofilms.^[^ ^47^ ^]^ However, despite the potent antimicrobial efficacy of Zn^2+^, their inherent cytotoxicity toward mammalian cells necessitates precise modulation of Zn^2+^ release kinetics. Consequently, achieving a controlled release profile is an essential prerequisite to ensure both biocompatibility and sustained antimicrobial efficacy.

In addition to the antibacterial effects of Zn^2+^, zinc oxide (ZnO) formed during the degradation of Zn‐based metals serves as an additional antimicrobial mechanism. When activated in aqueous environments, ZnO generates hydroxyl radicals (·OH), which can further react to produce hydrogen peroxide (H_2_O_2_) and other ROS. These ROS induce oxidative stress, leading to intracellular damage, such as DNA strand breaks, lipid peroxidation, and protein denaturation, ultimately resulting in bacterial death.^[^ ^91^ ^]^ Notably, hydroxyl radicals are the most potent ROS, capable of reacting indiscriminately with virtually all cellular molecules. Furthermore, two hydroxyl radicals (·OH) can react to form hydrogen peroxide (H_2_O_2_), perpetuating the oxidative cascade.^[^ ^92^ ^]^ Beyond that, corrosion products, such as zinc phosphate formed during the degradation of Zn‐based metals may augment the material's antibacterial efficacy.^[^ ^93^ ^]^ These byproducts exhibit intrinsic biocidal activity through mechanisms including pH modulation, ionic disruption of bacterial membranes, or synergistic interactions with Zn^2+^ released from the substrate. Therefore, under the synergistic effects of the aforementioned mechanisms, Zn‐based metals exhibit robust antibacterial activity, positioning them as promising candidates for barrier membrane applications.

### 3.6 Promoting Effect on Wound Healing

**Figure 9:**
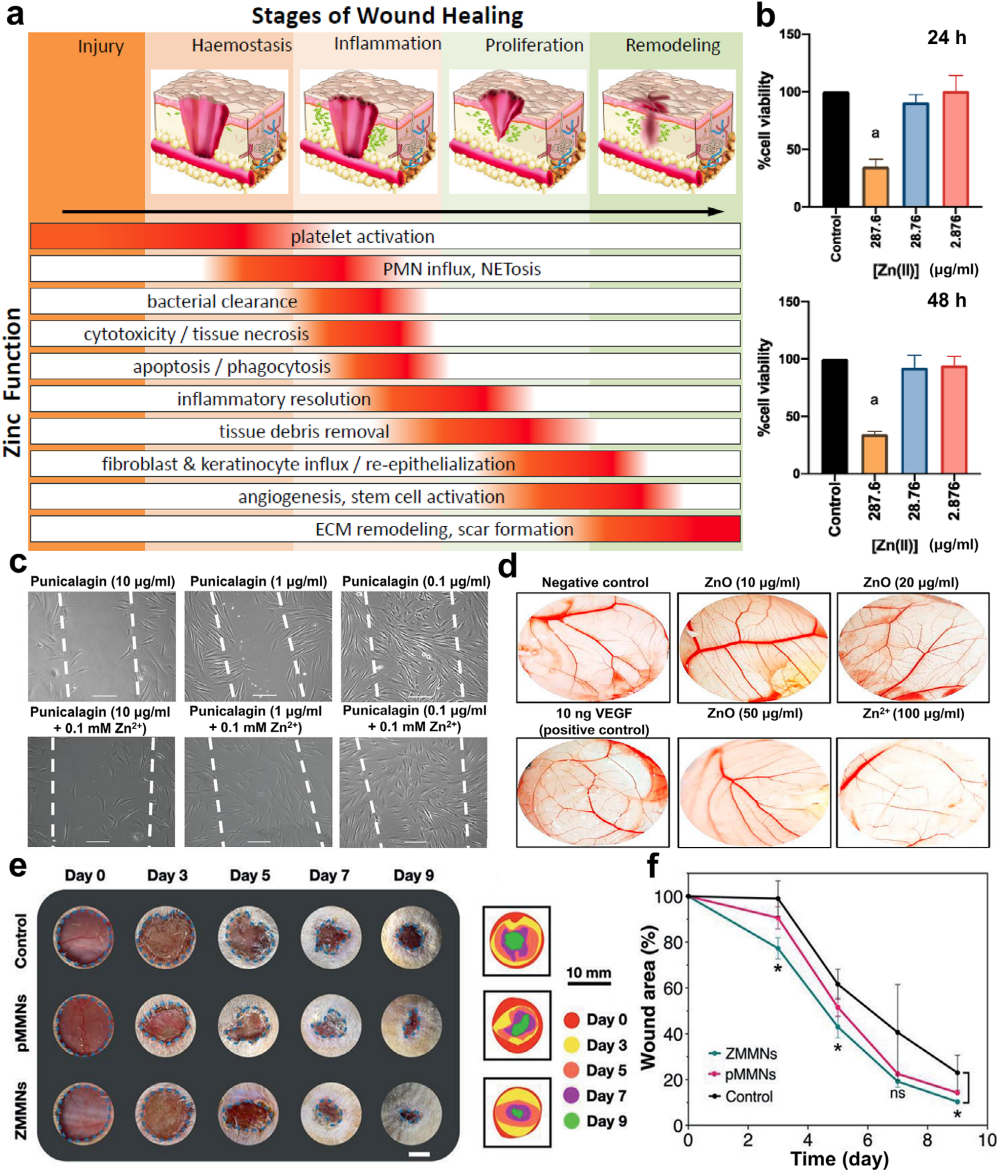
Assessment of Zinc‐based Barrier Membranes: Biotherapeutic Efficacy in Wound Repair and Tissue Regeneration Mechanisms. a) Zn in wound healing: mechanistic roles and stage‐specific regulatory functions across tissue repair phases. Reproduced with permission.^[^ ^95a^ ^]^ Copyright 2018, MDPI. b) Effects of Zn^2+^ gradients (2.876 µg mL^−1^ (0.01 mm) to 287.6 µg mL^−1^ (1 mm)) on human gingival fibroblasts: concentration‐dependent modulation of viability and proliferation dynamics. c) Spatiotemporal remodeling of HGFs: punicalagin dose‐response (0.1–10 µg mL^−1^) and Zn^2+^ synergy (0.1 mm) revealed through 48‐h optobiological monitoring (White dashed lines show original scratch wounds at 0 h, and scale bar = 100 µm). Reproduced with permission.^[^ ^104^ ^]^ Copyright 2018, MDPI. d) Ex‐vivo angiogenic profiling via chicken chorioallantoic membrane (CAM) assay: Comparative evaluation of untreated controls, VEGF (10 ng), ZnO nanorods (10–50 µg mL^−1^), and Zn^2+^ (100 µg mL^−1^). Reproduced with permission.^[^ ^108^ ^]^ Copyright 2021, Frontiers. Multimodal wound evaluation: e) Macroscopic imaging, topographic schematics, and f) closure metrics across treatment cohorts (Control, pMMNs (pure MeHA microneedles), ZMMNs (ZIF‐8@MeHA microneedles)). Reproduced with permission.^[^ ^107^ ^]^ Copyright 2021, Wiley‐VCH GmbH.

The barrier membrane is typically positioned between the bone defect area and the gingival soft tissue flap. To prevent postoperative infection and enhance the membrane's osteogenic efficacy in bone regeneration, the barrier membrane material should possess significant wound‐healing promotion capabilities.^[^ ^94^ ^]^ Generally, wound healing represents a complex, multistage physiological process that progresses through four interdependent yet temporally distinct phases (as shown in **Figure** **9**a): 1) Fibrin clot formation for hemostasis (initiated within seconds to 1 h postinjury); 2) Inflammatory response activation (unfolding from minutes to days); 3) Proliferative phase featuring cellular multiplication, re‐epithelialization, granulation tissue development, and neovascularization (commencing 18–24 h postinjury and persisting for days to weeks), and 4) Remodeling phase characterized by matrix reorganization and scar maturation (initiating 5–7 days post‐trauma and potentially continuing for months to years).^[^ ^95^ ^]^ This orchestrated sequence involves precise spatiotemporal coordination of diverse biological elements including soluble mediators (ROS, chemokines, cytokines, and growth factors), extracellular matrix (ECM) restructuring, and intricate cellular interactions among platelets, immune cells, resident keratinocytes, endothelial cells, fibroblasts, epithelial populations, and stem cell lineages.^[^ ^96^ ^]^

Zn^2+^ and ZnO are released when Zn‐based barrier membranes are gradually corroded, and they are crucial for regulating wound healing.^[^ ^95b^ ^]^ Metalloenzyme‐dependent processes during epithelial regeneration are crucially modulated by Zn as a cofactor for tissue‐repair‐related proteins and an essential constituent of metalloenzymes.^[^ ^97^ ^]^ Clinically, Zn deficiency or dyshomeostasis is pathologically linked to compromised healing outcomes and dermatopathological sequelae.^[^ ^98^ ^]^ First, Zn is closely linked to hemostasis, influencing plasma clotting factors, platelet aggregation, and their interactions; Zn^2+^ deficiency is associated with prolonged bleeding and abnormal platelet aggregation. Additionally, Zn plays a critical role in regulating inflammatory cell activity, particularly influencing neutrophil migration, phagocytosis, and nicotinamide adenine dinucleotide phosphate (NADPH)‐oxidase‐dependent ROS production for pathogen clearance; its deficiency or excess wound disrupt macrophage polarization (promoting M1 over M2) and impair B‐lymphocyte function, antibody production, and wound healing.^[^ ^99^ ^]^ Studies demonstrate that Zn supplementation can reverse neutrophil chemotaxis deficits caused by deficiency, though improper levels exacerbate immune dysregulation.^[^ ^100^ ^]^ Moreover, Zn acts as a critical cofactor in granulation tissue formation by signaling collagen/extracellular matrix deposition during the proliferation phase, and enhances keratinocyte migration through upregulated integrin expression to promote epidermal re‐epithelialization.^[^ ^37^, ^101^ ^]^ More importantly, Zn can regulate matrix metalloproteinases (MMP, Zn‐dependent enzyme) balance via tissue inhibitors of metalloproteinases (TIMPs), ensuring proper wound healing by preventing fibrosis or impairment.^[^ ^102^ ^]^

Indeed, recent studies on Zn's role in wound healing have garnered significant attention. Xu et al. recently engineered Zn‐incorporated poly(ionic liquid) (PIL) membranes, showcasing their potent antibacterial properties, excellent biocompatibility, and promising clinical applicability for wound dressing applications.^[^ ^103^ ^]^ Celiksoy et al. investigated the effects of varying Zn^2+^ concentrations on HGF cellular viability and proliferation.^[^ ^104^ ^]^ It was demonstrated that elevated Zn^2+^ levels (1 mm) significantly reduced cell viability, whereas lower concentrations (0.01–0.1 mm) did not impair cellular activity (Figure 9b). Additionally, the experimental data revealed that cotreatment with punicalagin (0.1–10 µg mL^−1^) and Zn^2+^ (0.1 mm) significantly boosted fibroblast migration speed and cumulative travel distance relative to punicalagin monotherapy (Figure 9c). This synergistic effect underscores Zn's dual role in activating anti‐inflammatory pathways and accelerating oral mucosal healing. Moreover, as the main degradation products of Zn‐based metals, ZnO acts as a pivotal wound healing enhancer by sustaining a localized 4–5‐fold increase in bioactive Zn^2+^, directly accelerating epithelialization, activating MMP‐mediated debridement of necrotic tissue, and upregulating tissue‐remodeling genes to boost collagen deposition and fibroblast activity.^[^ ^95a^ ^]^ Preclinical and clinical studies confirm the efficacy of ZnO: it reduces healing time by 30% in full‐/partial‐thickness wounds (rodent/porcine models), improves chronic venous ulcer repair, and supports advanced dressings (e.g., ZnO‐alginate, Zn‐saline) through dual antibacterial and regenerative mechanisms;^[^ ^37^, ^105^ ^]^ however, systemic toxicity risks, such as copper deficiency, nanoparticle‐induced hepatotoxicity, require dosage monitoring.^[^ ^106^ ^]^ As illustrated in Figure 9d, ZnO nanorods exhibit concentration‐dependent angiogenic effect. Low doses (10–20 µg mL^−1^) triple vascular growth metrics, rivaling VEGF efficacy, while higher doses (50 µg mL^−1^) or free Zn^2+^ inhibit neovascularization, highlighting ZnO's dual therapeutic potential in tissue repair and pathological angiogenesis control. Moreover, Yao et al. engineered microneedles (MNs) using methacrylated hyaluronic acid (MeHA) encapsulated with Zn‐metalorganic frameworks (Zn‐MOFs), showing that the sustained Zn^2+^ release from Zn‐MOFs accelerates epithelial regeneration and neovascularization, significantly enhancing wound healing (Figure 9e,f).^[^ ^107^ ^]^

## 4 Recent Advances in the Development of Zn‐Based Alloy Membranes

Biodegradable Zn‐based metals have emerged as promising candidates for barrier membrane materials, although this concept is still relatively novel and current research is in its early stages. As a result, researchers are concentrating on four main approaches to enhance the performance of Zn‐based barrier membranes: alloying, surface modification treatments, the development of Zn‐based composite barrier membranes, and additive manufacturing techniques.

### 4.1 Novel Zn‐Based Alloy Membrane Fabricated by Alloying Methods

While pure Zn barrier membranes exhibit favorable biocompatibility, our preliminary studies revealed comparable osteogenic performance to pure Ti barrier membranes after 10 week implantation in a rabbit cranial defect model.^[^ ^18a^ ^]^ To enhance the bioactivity of Zn‐based barrier membranes, researchers have prioritized alloying Zn with physiologically active elements such as Mg, Cu, Ag, and Li during material design.^[^ ^109^ ^]^ As summarized in **Table** **2**, recent advances in pure Zn and Zn alloys for GBR membranes are thoroughly documented. Current findings confirmed that the incorporation of alloying elements generally leads to substantial enhancement in mechanical properties, particularly strength and ductility.^[^ ^11^ ^]^ Actually, this approach often results in secondary phase formation, such as the Mg_2_Zn_11_ phases in Zn‐Mg alloys.^[^ ^110^ ^]^ These secondary phases typically refine the Zn matrix microstructure, improving material strength and ductility via grain refinement. Additionally, they hinder dislocation movement during plastic deformation, contributing to consistent improvements in the mechanical strength of Zn alloys.^[^ ^111^ ^]^ However, these secondary phases can create electrochemical potential differences with the matrix, resulting in microgalvanic corrosion that accelerates substrate degradation. To date, contemporary alloying strategies have evolved from single‐element to multi‐element systems, incorporating essential trace elements relevant to human physiology. This development aims to enhance the multifunctional bioactivity of Zn‐based membranes, promoting osteogenesis, immunomodulation, and antimicrobial effects.

**Table 2:** A comprehensive summary of recent developed biodegradable Zn‐based metals as GBR membrane.

Materials^a)^	Fabrication method	YS [MPa]	UTS [MPa]	El [%]	In vitro degradation rate [mm/year^−1^]		In vitro biological effects	Pathological model	Implant size [mm]	In vivo therapeutic effects	Refs.
					Immersion test	Electrochemical					
Pure Zn (Without pores) Pure Zn (300 µm pores) Pure Zn (1000 µm pores)	Hot rolling + Laser cutting	92.2 47.6 41.5	108.0 52.1 48.7	42.8 13.6 7.8	0.042 0.046 0.052		i) Diluted alloy extracts showed favorable cytocompatibility toward MC3T3‐E1 cells. ii) 100% alloy extracts exhibited significant cytotoxicity.	Rat calvarial critical‐sized bone defect model	9 × 9× 0.03	After 10 weeks, 300 µm‐pore Zn membranes showed enhanced new bone formation (slightly higher than Ti controls).	[18a]
Pure Zn	Extruded				0.031	0.029	i) Acceptable cytocompatibility to HGF cells ii) Exhibited potent antibacterial activity against *P. gingivalis *				[29c]
Alloying method											
Zn‐0.6Cu thin sheet	Extruded	93.0	108.4	121.0	0.046	0.158	i) Sound biocompatibility and bioactivity (osteogenesis, antibacteria, tissue recovery). ii) No interference with X‐ray and MRI.	Rat calvarial critical‐sized bone defect model	Φ 0.5 × 0.2	The newly formed bone tissues were in direct contact with the Zn‐0.6Cu implant, showing good in vivo biocompatibility.	[112]
Zn‐0.3Ca sheet	Bottom circulating water‐cooled casting (BCWC) + Rolling	146.0	174.0	49.0	0.154	0.192	i) Favorable cytocompatibility and enhanced osteogenic differentiation performance in MC3T3‐E1 cells. ii) Dual antibacterial activity against both *S. aureus* and *E. coli*.				[113]
Zn‐1Cu plates Zn‐2Cu plates Zn‐4Cu plates	As‐rolled As‐rolled As‐rolled	236.5 278.4 327.1	294.5 327.3 393.3	44.6 48.1 38.8		0.06 0.13 0.13	i) Enhanced metabolic activity in mouse fibroblast (L929), human immortalized cranial periosteal (TAg), and osteosarcoma (Saos‐2) cell lines. ii) Inhibited biofilm formation of mixed oral bacteria.				[114]
Zn‐0.5Fe Zn‐0.5Fe Zn‐0.5Fe	Powder sintering Hot extruded Hot rolling	101.3 110.2	100.5 150.9 168.8	0.5 19.9 16.3		0.146 0.125 0.115	i) Sound cytocompatibility to MC3T3‐E1. ii) The cytocompatibility presents a dose‐dependent relationship.				[115]
Zn‐0.5Cu Zn‐0.5Cu‐0.1Fe Zn‐0.5Cu‐0.2Fe Zn‐0.5Cu‐0.4Fe	Extruded Extruded Extruded Extruded	113.1 115.7 152.3 182.1	164.2 176.0 202.3 240.1	35.5 43.9 41.2 20.5	0.035 0.037 0.042 0.050		i) Improved metabolic activity in mouse fibroblast (L929), human immortalized cranial periosteal (TAg), and osteosarcoma (Saos‐2) cell lines. ii) High antibacterial activity against *S. gordonii* and mixed oral bacteria.				[47]
Zn‐0.5Cu‐0.2Fe sheets	As‐rolled				62.54 [µm cm^−2^ day^−1^]		i) Sound cytocompatibility to RAW264.7, HUVEC, and MC3T3‐E1 cell lines. ii) Appropriate hemocompatibility.				[35a]
Zn‐4Ag sheets Zn‐4Ag‐0.1Sc sheets	Hot rolled Hot rolled	181.8 202.0	222.2 260.5	52.1 72.7	0.025 0.037	0.278 0.559	i) Good cytocompatibility to both MC3T3‐E1 and MG‐63 cells lines. ii) High antibacterial activity against *S. aureus* strains.	Rat calvarial critical‐sized bone defect model		*i*. Sound in vivo antibacterial activity and anti‐inflammatory ability. *ii*. Good ability to guide bone repair in vivo.	[116]
Zn‐Mg‐Fe plates and screws					0.095 (In vivo)			Beagle canine mandibular fracture model	Four‐hole plates (1 mm thick, 24 mm long).	Compared to the PLLA and Ti groups, the Zn alloy group demonstrated a greater capacity for osteogenesis and bone remodeling.	[56b]
Zn‐0.8Li Zn‐0.8Li‐0.2Mg Zn‐0.8Li‐0.2Ag	As‐rolled As‐rolled As‐rolled	183.5 253.7 196.2	238.1 341.3 254.7	75.0 30.6 97.9		0.120 0.170 0.110	i) The cytotoxicity of these extracts of Zn‐Li‐Ag alloy was of Grade 0‐1. ii) The Zn‐Li‐Ag alloy is innocuous.				[117]
Zn‐Ti‐Cu‐Ca‐P	Powder sintering	214.0				0.180	Good biocompatibility to Vero cells.				[118]
Zn‐0.5Ti‐0.5Fe Zn‐0.5Ti‐0.5 Mg	Powder sintering		145.3 164.4	30.2 19.3		0.181 0.199	i) Enhanced mechanical performance. ii) Promoted corrosion rate. iii) Sound cytocompatibility to MC3T3‐E1.	New Zealand rabbit skull defect model	Φ 8× 0.1	Effective in vivo guided bone regeneration	[119]
Zn‐0.3Fe‐0.05 Mg	Powder sintering + Multiple rolling processes		294.07	20.67		0.18	i) Promoted osteogenic differentiation. ii) Stimulated macrophage secretion of anti‐inflammatory cytokines iii) Sound angiogenesis ability.	Mouse air pouch model, New Zealand rabbit skull defect model	Φ 8× 0.06	Modulate osteoimmunology and promote early vascularized bone regeneration.	[120]
Zn‐0.5Fe‐0.05Mg Zn‐0.5Mg‐0.05Fe	Powder sintering		331.2 352.2	25.8 20.2		0.180 0.210	i) Enhanced mechanical performance. ii) Promoted corrosion rate. iii) Sound cytocompatibility to MC3T3‐E1.	New Zealand rabbit skull defect model	Φ 8× 0.1	Effective in vivo guided bone regeneration	[121]
Surface modification											
Pure Zn (Sa < 0.1 µm) Pure Zn (Sa: 0.5–1.0 µm) Pure Zn (Sa > 1.0 µm)	Mechanical grinding using silicon carbide grinding paper				0.009 0.010 0.005		i) Cytotoxicity assays revealed no significant difference between pure Zn with different surface roughness patterns. ii) Macrophages tended to shift toward a proinflammatory state when exposed to nanoscale pure Zn. iii) Micrometer and submicrometer‐scale surfaces exhibited stronger antibacterial effects than the nanoscale surface.				[122]
Porous Zn‐3Cu‐1Mg Ca‐P coated porous Zn‐3Cu‐1Mg	Powder sintering + Infiltration casting + Chemical deposition						i) Promote BMSCs activity and proliferation ii) The osteogenic differentiation of BMSCs was enhanced.	A rabbit cranial bone defect model	Φ 10 × 2	New bone formation in the coated sample group was significantly greater than that in pure Zn and Ti alloy groups.	[123]
Zn‐1Cu‐0.1Ti ZnP‐Coated Zn‐1Cu‐0.1Ti	Hot rolled + Chemical deposition	253.0	299.0	33.6	0.039 0.027	0.440 0.060	i) Favorable cytocompatibility to MC3T3‐E1 cells and MG63 cells. ii) Sound osteogenic differentiation ability. iii) Good antibacterial activity against *S. aureus*.	Rat calvarial critical‐sized bone defect model	Custom size	Post 3‐month in vivo implantation, the ZnP coating markedly enhanced the material's osteogenic capacity, facilitating bone regeneration.	[124]
Pure Zn Mg‐MOF coated Zn Mg/1Cu‐MOF coated Zn Mg/3Cu‐MOF coated Zn Mg/5Cu‐MOF coated Zn	Extruded + Hydrothermal reaction				0.032 0.065 0.104 0.132 0.146	0.035 0.046 0.076 0.074 0.086	i) Good cytocompatibility to MC3T3‐E1 cells and HUVEC cells. ii) Sound osteogenic differentiation, angiogenesis and wound healing ability. iii) Sufficient antibacterial activity against *S. aureus* and *E. coli*.	Mouse Subcutaneous Infection Mode	Φ 10 × 1	*i*. Mg/Cu1 samples show a sound in vivo bacteriostatic activity against *S. aureus*. *ii*. Mg/Cu1 samples present good angiogenesis and anti‐inflammatory ability.	[125]
Zn‐0.5Li Fe/ZnP coated Zn‐0.5Li	Extruded + Chemical deposition					0.019 0.002	i) Modulate the degradation behavior of substrate. ii) Good cytocompatibility to MC3T3‐E1.				[126]
Pure Zn MXene‐coated Zn (0.5 mg mL^−1^) MXene‐coated Zn (1.0 mg mL^−1^) MXene‐coated Zn (1.5 mg mL^−1^)	Extruded + Dip coating				0.019 0.007 0.005 0.004	0.385 0.174 0.147 0.101	i) Modulate the initial degradation behavior. ii) Promoted osteogenic differentiation, enhanced HGF cell migration, and stimulated macrophage secretion of anti‐inflammatory cytokines iii) Sufficient antibacterial activity against *S. aureus* and *E. coli*.	Mouse Subcutaneous Infection Mode	Φ 10 × 1.5	*i*. MXene‐coated Zn show a sound in vivo bacteriostatic activity against *S. aureus*. *ii*. MXene‐coated Zn present good anti‐inflammatory ability.	[127]
Composites design											
Pure Zn mesh (ZM) Single sided complex PCL‐CS Zn mesh membrane (SSZM) Double sided complex PCL‐CS Zn mesh membrane (DSZM)	As rolled + Electrospinning		19.4 24.7 25.6	19.8 16.6 16.1		0.387 0.366 0.017	i) Promote osteoblast activity and adhesion. ii) Strong osteogenic differentiation potential. iii) Sufficient antibacterial activity against *S. aureus*.	Rat maxillary defect model	13 × 25 × 0.1 mm	*i*. The newly formed bone in the DSZM defect region has a more pronounced bone height and a bone structure. *ii*. Favorable in vivo biosafety.	[128]
Zn MAO‐Zn Zn/Poly(lactic acid) (PLA) composite films	Micro‐arc oxidation + Hot pressing		27.95 27.79 55.57			0.383 1.245 NA					[129]
Additive manufacturing											
Porous Zn Porous Zn loaded with Chitosan/gelatin cryogel (CSG).	Electrophoretic deposition + Laser powder bed fusion		5.60 8.21	6.45 9.22		1.810 0.190	i) Favorable cytocompatibility and osteogenic capability. ii) Long‐acting antibacterial activity against *S. aureus*.				[130]

^a)^ YS: Yield strength (MPa), UTS: Ultimate tensile strength (MPa), El: Elongation (%), and NA: Not available.

Despite the widespread use and effectiveness of alloying, current approaches to modifying Zn‐based barrier membranes have notable limitations. Key issues include: 1) ‌Insufficient mechanistic coherence‌ across alloy systems, lacking discernible structure–property relationships that unify different alloys and systematic composition optimization within homologous systems; 2) ‌Limited multifunctional biocompatibility‌, as alloying alone often fails to provide comprehensive biological functionalities, such as robust angiogenic, osteogenic, and antibacterial properties; 3) Inadequate defect‐specific structural adaptation‌, where current designs do not incorporate morphological customization aligned with alveolar bone defect geometries. Consequently, researchers are increasingly shifting from alloy‐based fabrication of Zn barrier membranes toward advanced strategies, such as surface modification, composite fabrication, and additive manufacturing, to develop next‐generation Zn‐based membrane materials.

### 4.2 Surface Modification on Zn‐Based Alloy Membrane

Compared to alloying strategies, surface modification represents a well‐established and economically viable processing paradigm that enables rational design of both compositional and topographical surface features tailored to application‐specific biological requirements. This microstructural engineering approach allows systematic integration of multiplexed biofunctional characteristics onto bulk materials without compromising their intrinsic properties. Generally speaking, the surface coatings of biodegradable metals are primarily categorized into conversion coatings (ion implantation, microarc oxidation, chemical conversion, layered double hydroxides, etc.) and deposited coatings (bioactive ceramics, synthetic polymers, natural polymers, oxide ceramics, etc.).^[^ ^131^ ^]^ Given that the two most critical properties of GBR membranes are bone repair performance and antibacterial activity, some organic/inorganic coatings, as summarized in Table 2, have been engineered on Zn‐based membranes to endow the material with synergistic osteogenic and antibacterial functionality. For example, Tong et al. successfully prepared ZnP coatings on a Zn‐1Cu‐0.1Ti mesh.^[^ ^124^ ^]^ Both in vitro and in vivo results demonstrated that the membrane not only exhibited excellent guided bone regeneration capability but also displayed antibacterial activity and antibiofilm efficacy against *S. aureus* bacteria. Besides, Li et al. recently fabricated pure Zn barrier membranes with varying surface roughness, and found that submicrometer‐scale Zn surfaces optimize degradation control and biocompatibility for bone regeneration, while nano‐ and micrometer‐scale counterparts exhibit accelerated corrosion and reduced antibacterial efficacy, establishing submicron Zn as the ideal barrier membrane material.^[^ ^122^ ^]^ All coating methodologies offer distinct advantages and limitations, and experimental conditions can significantly influence key coating characteristics, such as thickness, composition, and density. This variability necessitates careful evaluation for Zn‐based bone implants.^[^ ^132^ ^]^ While surface modification enables rapid functionalization of Zn‐based barrier membranes with customized bioactivity, ensuring long‐term stability remains a critical challenge that directly affects the sustained therapeutic efficacy of these implants. Consequently, recent research has increasingly shifted its focus from surface modification alone to composite‐based strategies aimed at enhancing membrane performance.

### 4.3 Zn‐Based Alloy Composite Membrane

In recent years, Zn‐based composites have garnered significant research attention as a promising approach to address the inherent limitations of pure Zn (tensile strength: ≈120 MPa; elongation: ≈2%) by simultaneously enhancing mechanical performance and biological functionality.^[^ ^133^ ^]^ The incorporation of reinforcements via cost‐effective manufacturing routes effectively strengthens Zn matrix while maintaining scalability for clinical applications. Current strategies predominantly utilize ceramic particles, carbon fibers, or metallic particulates as reinforcing phases in Zn‐based composites.^[^ ^134^ ^]^ Current research on Zn‐based composites prioritizes in situ synthesis as the primary methodology for fabricating thermodynamically stable reinforcements (e.g., carbides, metallic oxides) with homogeneous dispersion and strong interfacial bonding. This advanced approach complements conventional mechanical alloying techniques while synergizing with emerging strategies such as heteroaggregation and graphene reinforcement.^[^ ^135^ ^]^ While in situ methods mitigate agglomeration and wettability issues, challenges persist in achieving high nanofiller dispersion (e.g., ceramic floatation in casting, contamination risks in powder metallurgy), optimizing cost‐effective scalability, and addressing mechanical‐performance gaps (e.g., strength‐ductility trade‐offs).^[^ ^136^ ^]^ However, current research on Zn‐based composites as GBR membranes remains extremely scarce, as shown in Table 2. Yan et al. recently developed a Zn‐based composite bilayer membrane (ss‐HMC/Zn) by integrating metallic Zn and hierarchical mineralized collagen (HMC), addressing limitations of current GBR membranes by combining mechanical durability, controllable Zn^2+^ release, and self‐induced osteogenesis.^[^ ^128^ ^]^ The composite synergistically combines the spatiotemporal barrier function of the Zn layer with the bone‐mimetic nanostructure of HMC, enhancing osteoinduction, angiogenesis, and immunomodulation. This approach presents a promising strategy for maxillofacial bone regeneration without the need for adjunctive interventions. Cai et al. also fabricated a Zn‐based composite film (MAO‐Zn/PLA) through microarc oxidation and hot‐pressing, which achieved a 17.7% increase in mechanical strength, solid interfacial bonding via mechanical interlocking and electrostatic attraction, and degradation rates acceleration (0.383–1.245 µm year^−1^), establishing a strategic approach to optimize GBR membranes.^[^ ^129^ ^]^ While Zn‐based composites can inherently enhance the physicochemical and biological properties of barrier membranes, they do not address the clinical challenge of patient‐specific alveolar ridge defects, which required tailored membrane geometries and dimensions. This limitation highlights the need for additive manufacturing to produce customizable Zn‐based barrier membranes.

### 4.4 Additively Manufactured Zn‐Based GBR Membrane

The additive manufacturing (AM) of biodegradable Zn metals is still in its early stages, yet it offers revolutionary potential for dental implants due to its ability to enable personalized design, create complex structures, and control degradation rates. Significant advancements have been made in the field of AM for biodegradable Zn‐based metals, particularly through the refinement of key AM techniques tailored for Zn materials. Prominent methods include ‌Laser Powder Bed Fusion (L‐PBF)‌, ‌Binder Jetting (BJ)‌, and ‌Direct Energy Deposition (DED)‌, each possessing advantages and limitations for Zn processing.^[^ ^137^ ^]^ Among these, L‐PBF‌ is widely regarded as the most adopted technique for Zn‐based biomaterials, providing exceptional precision and design flexibility that allows for the fabrication of complex porous structures with customized mechanical and degradation properties. However, challenges such as Zn's tendency to vaporize during laser melting necessitate optimized gas circulation systems to minimize fume interference.^[^ ^138^ ^]^ In contrast, ‌BJ‌ encounters limitations related to achieving adequate densification and mechanical integrity, while ‌DED‌ is better suited for larger components but lacks the precision required for intricate biomedical implants.^[^ ^139^ ^]^ Recent advancements in L‐PBF have successfully produced Zn alloys (e.g., Zn‐Mg, Zn‐Li) and composites (e.g., Zn‐RGO, Zn‐SiC), which demonstrated enhanced mechanical strength and biocompatibility.^[^ ^140^ ^]^ These improvements are supported by rigorous studies on powder preparation, process parameter optimization, and post‐treatment techniques.

For example, Liu and colleagues recently developed a novel biodegradable GBR membranes using synergizing laser powder bed fusion (LPBF) 3D printing with electrophoretic assembly technology. The membrane comprises a porous Zn substrate with optimized structural integrity and stress distribution; a conformal chitosan/gelatin cryogel coating was then applied to the 3D‐printed framework, significantly enhancing mechanical robustness, slowing biodegradation rates, and preventing fibroblast penetration.^[^ ^130^ ^]^ All the above designs made the new composite membrane exceptional dual functionality‐promoting osteogenesis and resisting bacterial infection, thereby showcasing the viability of LPBF‐based 3D printing for creating patient‐specific implants with multifunctional capabilities in bone defect repair. Although current studies on additive‐manufactured Zn‐based membranes are limited, future research may explore the synergistic integration of alloying, surface modification, and composite material design to engineer next‐generation Zn‐based membranes with multidimensional bioactive functionalities.

## 5 Shortcomings Faced with Zn‐Based BM Membrane

**Figure 10:**
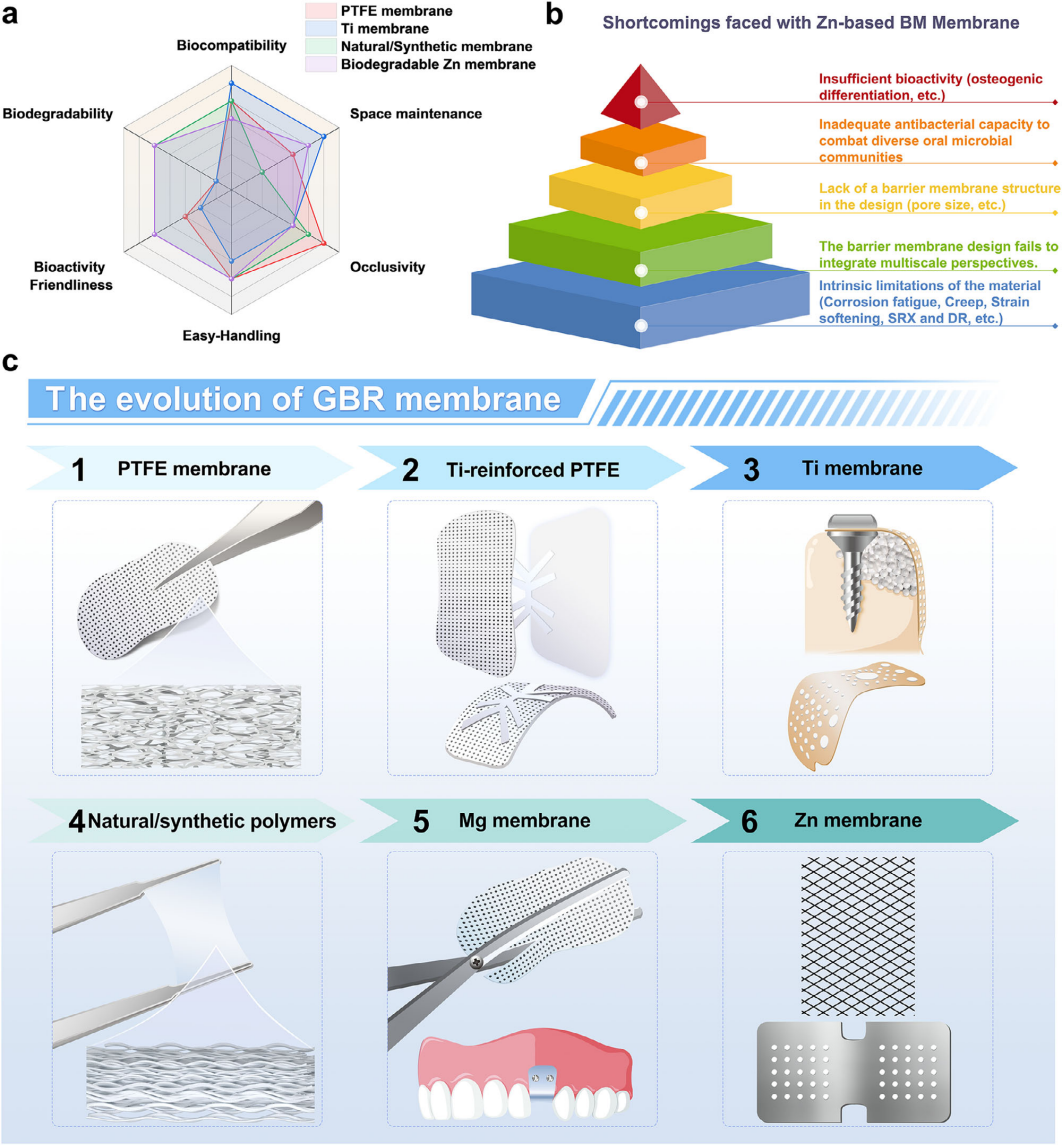
Comprehensive performance comparison of Zn‐based BM membranes. a) Holistic performance evaluation of different barrier membranes. b) Shortcomings faced with Zn‐based BM membranes, and c) Temporal evolution of GBR membranes.

As noted, while Zn and Zn‐based metals have been validated as promising candidates for barrier membrane applications, critical challenges spanning biological compatibility (e.g., inflammatory response, osteogenic balance) and material science (e.g., degradation control, mechanical stability) must be systematically addressed to bridge the translational gap from bench to bedside (**Figure** **10**a).

From a biological perspective, the biological function of barrier membranes is primarily associated with their Zn^2+^ release. After implantation, the upper region of the membrane is subjected to tensile stress, while the lower region experiences compressive stress, leading to distinct matrix degradation behaviors between these two regions. Additionally, due to the differentiated functional characteristics of the inner and outer layers, variations in degradation rates and patterns are required for the material surfaces. The primary function of barrier membranes is to provide stable mechanical support and spatial stability for bone defect areas. Consequently, it is essential to elucidate the differences in degradation behavior and mechanisms between the inner and outer layers under bending deformation, as well as their distinct biological impacts. Furthermore, there is currently a lack of systematic assessment of the in vivo biological effects of barrier membrane materials. Most existing studies evaluate the feasibility of materials as barrier membranes based on their mechanical properties, in vitro degradation behavior, and biological effects. However, longitudinal in‐depth research targeting optimized Zn‐based alloy compositions for specific clinical applications, such as alveolar bone defects, remains insufficient. Additionally, there is a critical knowledge gap in understanding how multiple metal ions released during the in vivo degradation of Zn‐based barrier membranes influence cellular behaviors and functions across different cell types (e.g., osteoblasts, macrophages, and vascular‐forming cells), as well as the histological responses throughout the complete bone repair process in defect areas. The spatiotemporal regulation of multi‐ion biological effects and their systemic impact on bone regeneration mechanisms require further elucidation.

From a materials science perspective, the interplay between localized stress and the degradation kinetics of barrier membranes must be rigorously evaluated to ensure their functional adaptation to diverse alveolar bone defect geometries and dynamic oral environments, particularly under cyclic masticatory loading. Although studies have begun to characterize SCC in barrier membranes, the physiological complexity of in vivo conditions raises concerns about concurrent corrosion fatigue (CF) mechanisms. Prior research by Li et al. demonstrated that the fatigue lifetimes of Zn‐0.8Li and Zn‐2Cu‐0.8Li alloys in simulated body fluid (SBF) were significantly shorter than those in ambient air, with a marked decline as stress amplitude increased, underscoring CF's detrimental impact on mechanical integrity under clinically relevant stressors.^[^ ^141^ ^]^ Additionally, some inherent material limitations of Zn must be systematically addressed prior to its clinical deployment: 1) Static recrystallization (SRX) and dynamic recovery‌ (DR): Zn's low recrystallization temperature (≈10 °C) promotes dynamic recovery under deformation, leading to localized strain concentration and nonuniform mechanical failure.^[^ ^142^ ^]^ This strain‐softening behavior, characterized by rapid postyield stress decline and necking, compromises structural integrity in load‐bearing oral environments. 2) Creep susceptibility‌: Under physiological temperature (37 °C) and cyclic masticatory loads (tension/compression), Zn alloys exhibit accelerated creep deformation due to thermally activated dislocation glide, posing risks of dimensional instability.^[^ ^143^ ^]^ 3) Natural aging at ambient conditions‌: Low melting point and Mg‐containing precipitates (e.g., Mg_2_Zn_11_ in Zn‐Mg alloys) drive progressive embrittlement, with elongation loss exceeding 50% in Zn‐0.05Mg after 1‐year storage.^[^ ^110^, ^144^ ^]^ Recent studies indicate that ternary alloying (0.1Mn/0.5Cu) may mitigate aging by stabilizing solid solutions and suppressing precipitate coarsening.^[^ ^145^ ^]^ 4) Strain softening: Post‐UTS stress reduction induces mechanical instability, creating vulnerability to crack initiation under oral function. This is exacerbated by SRX‐induced grain boundary migration during cyclic loading.^[^ ^146^ ^]^ Consequently, systematic investigations into these critical challenges in Zn alloys warrant prioritized research focus (Figure 10b). Notably, the membrane's differential microenvironments at inner/outer interfaces, coupled with sustained masticatory stresses in oral applications, make the concurrent fulfillment of divergent biological requirements and enhanced SCC/CF resistance a critical research frontier.

## 6 Future Prospects

The progressive evolution of GBR membrane technology has positioned degradable Zn‐based membranes as a pivotal research frontier (Figure 10c), driven by their unique capacity to synergize mechanical durability, tunable degradation kinetics, and osteoimmunomodulatory functionality in craniomaxillofacial applications. Considering the existing advantages and limitations of current Zn‐based barrier membrane materials, future developments in Zn‐based barrier membranes could focus on the following directions:

1. Currently, the blood and body fluid transport functions of GBR membranes are often overlooked, resulting in the inability of nutrient‐ and protein‐rich body fluids to transfer from the outer side of the membrane to the internal bone defect area. This impedes metabolic activity and tissue regeneration, thereby reducing osteogenic efficiency. Moreover, the design principles governing pore structures in Zn‐based barrier membranes have not been systematically investigated, and their consequential impacts on both material properties and biological performance remain poorly understood. Consequently, developing Zn‐based GBR membranes that simultaneously achieve mechanical strength, barrier isolation, and effective directional nutrient transport remains a major challenge.
2. As barrier membranes are required to concurrently possess robust bone repair guidance capabilities and antibacterial activity, achieving an optimal balance between osteogenesis and antimicrobial efficacy remains a critical challenge. First, it is essential to precisely regulate Zn^2+^ degradation kinetics through approaches, such as microalloying, surface modification, and novel preparation methods (e.g., high‐pressure solid‐solution (HPSS) treatment, supersaturated solid‐solution treatment (SSST), nanoparticle‐reinforced composites prepared via roll‐bonding, and extrusion combined with caliber rolling).^[^ ^147^ ^]^ This modulation aims to maintain Zn^2+^ concentrations within the therapeutic window between effective antibacterial thresholds and osteogenic‐promoting levels. Second, the localized Zn^2+^ concentration gradient near the membrane surface frequently exceeds physiological levels in surrounding body fluids, potentially compromising cellular adhesion morphology of critical cell types including osteoblasts and macrophages. Therefore, multiscale regulation of Zn‐based GBR membranes (spanning micro‐to‐macro dimensions) appears to be an effective strategy to address this biocompatibility‐structural paradox.
3. Current surface modification approaches and coating architectures for barrier membranes remain oversimplified, often failing to endow the materials with multifunctional biological properties while neglecting to differentiate the distinct functional requirements between the outer and inner layers. Future research should adopt a compartmentalized design strategy for Zn‐based barrier membranes, implementing stratified surface modifications tailored to their dual functional zones: the upper region (facilitating soft tissue healing, antibacterial action, and anti‐inflammatory response) and the lower region (promoting vascularization, osteogenesis, and localized immunomodulation).
4. When engineering novel Zn‐based barrier membranes, a hierarchical design paradigm should be implemented across ‌compositional engineering, surface topography engineering, hierarchical structural engineering‌, enabling holistic regulation of both ‌cellular dynamics‌ and ‌tissue‐level responses‌ in the peri‐membrane microenvironment. This multiscale material governance framework empowers Zn‐based membranes with ‌spatiotemporally programmable degradation kinetics‌ and ‌multidimensional biofunctionality‌.
5. In order to comply with the updated definition of biodegradable metals (total degradation in vivo with no residues and metabolizable byproducts that help tissue regeneration), it is imperative to develop fully (100%) biodegradable Zn‐based GBR membranes. Therefore, it is crucial to make sure that the material and its degradation products are completely (100%) biodegradable when constructing Zn‐based GBR membranes in the future, whether through alloying, surface modification, or other techniques.
6. The use of large animal models is scientifically essential. Porcine and ovine species demonstrate cranial dimensions, cortical thickness, and defect sizes that closely match human anatomy. While rodent/rabbit calvarial defects are typically under 8 mm, human clinical defects frequently surpass 10 mm, making large animals necessary for proper GBR membrane implantation and accurate simulation of osteogenic requirements. Their masticatory forces and biomechanical loading more faithfully reproduce human physiological conditions, allowing reliable assessment of membrane stability and bone formation under functional loads. Importantly, their extended healing periods and comparable immune responses (particularly in inflammation patterns and foreign body reactions) enable detection of chronic rejection risks that small animal models cannot identify.

## 7 Summary

Biodegradable Zn‐based alloys show promise for GBR membranes by overcoming the limitations of traditional materials, such as rapid degradation and poor mechanical strength. These alloys offer moderate degradation rates, biocompatibility, osteogenic activity via Zn^2+^‐mediated pathways, and antibacterial effects through membrane disruption and ROS generation. Alloying with elements like Mg, Cu, and Li as well as surface modifications, enhance mechanical stability, bioactivity, and degradation control. Additionally, additive manufacturing enables patient‐specific designs. However, challenges remain, including stress corrosion, creep, and the need to balance osteogenesis and antimicrobial efficacy, which needs to be addressed in future studies. Overall, Zn‐based barrier membranes present a biodegradable, multifunctional solution for improved bone regeneration in dental applications.

## Conflict of Interest

The authors declare no conflict of interest.
